# Cancer immune control dynamics: a clinical data driven model of systemic immunity in patients with metastatic melanoma

**DOI:** 10.1186/s12859-021-04025-7

**Published:** 2021-04-16

**Authors:** Harold P. Frisch, Allan Sprau, Virginia F. McElroy, James D. Turner, Laura R. E. Becher, Wendy K. Nevala, Alexey A. Leontovich, Svetomir N. Markovic

**Affiliations:** 1 Payload Systems Engineering Branch, Emeritus, NASA, Annapolis, MD USA; 2 Math for Medicine, Inc., Rochester, MN USA; 3 Retired Aerospace Consultant, Texas A&M University, College Station, TX USA; 4 Department of Oncology Research, Mayo Clinic, Rochester, MN USA; 5 Department of Biomedical Statistics and Informatics, Mayo Clinic, Rochester, MN USA; 6 Department of Medical Oncology, Mayo Clinic, 200 First Street SW, Rochester, MN 55905 USA

**Keywords:** Math modeling, Oncology, Melanoma, Systemic immunity, Peripheral blood biomarkers

## Abstract

**Background:**

Recent clinical advances in cancer immuno-therapeutics underscore the need for improved understanding of the complex relationship between cancer and the multiple, multi-functional, inter-dependent, cellular and humoral mediators/regulators of the human immune system. This interdisciplinary effort exploits engineering analysis methods utilized to investigate anomalous physical system behaviors to explore immune system behaviors. Cancer Immune Control Dynamics (CICD), a systems analysis approach, attempts to identify differences between systemic immune homeostasis of 27 healthy volunteers versus 14 patients with metastatic malignant melanoma based on daily serial measurements of conventional peripheral blood biomarkers (15 cell subsets, 35 cytokines). The modeling strategy applies engineering control theory to analyze an individual’s immune system based on the biomarkers’ dynamic non-linear oscillatory behaviors. The reverse engineering analysis uses a Singular Value Decomposition (SVD) algorithm to solve the inverse problem and identify a solution profile of the active biomarker relationships. Herein, 28,605 biologically possible biomarker interactions are modeled by a set of matrix equations creating a system interaction model. CICD quantifies the model with a participant’s biomarker data then computationally solves it to measure each relationship’s activity allowing a visualization of the individual’s current state of immunity.

**Results:**

CICD results provide initial evidence that this model-based analysis is consistent with identified roles of biomarkers in systemic immunity of cancer patients versus that of healthy volunteers. The mathematical computations alone identified a plausible network of immune cells, including T cells, natural killer (NK) cells, monocytes, and dendritic cells (DC) with cytokines MCP-1 [CXCL2], IP-10 [CXCL10], and IL-8 that play a role in sustaining the state of immunity in advanced cancer.

**Conclusions:**

With CICD modeling capabilities, the complexity of the immune system is mathematically quantified through thousands of possible interactions between multiple biomarkers. Therefore, the overall state of an individual’s immune system regardless of clinical status, is modeled as reflected in their blood samples. It is anticipated that CICD-based capabilities will provide tools to specifically address cancer and treatment modulated (immune checkpoint inhibitors) parameters of human immunity, revealing clinically relevant biological interactions.

**Supplementary Information:**

The online version contains supplementary material available at 10.1186/s12859-021-04025-7.

## Background

In recent years, new insights into the state of the systemic immunity in cancer patients suggest a constellation of multiple abnormalities in the immune system that have the potential to directly impact not only the clinical response to immune checkpoint inhibitors (ICI) therapy but also the natural history of the malignant disease [1]. It is becoming increasingly clear that the panoply of multiple aberrancies in the immune system of cancer patients likely represent a manifestation of a complex set of biological processes that require an interrogative approach capable of complex systems analyses that take into account multiple variably interdependent parameters (biomarkers). The scientific community is increasingly recognizing that to analyze such complex systems, a challenging interdisciplinary approach to create meaningful biological computational tools is urgently needed [2–7]. A team of medical oncologists/immunologists and systems engineers have come together to overcome these challenges by applying well-established mathematical algorithms and engineering knowledge of physical non-linear oscillations to biological oscillations of cells and cytokines in the immune system [8–12]. The product of this multi-year collaboration is the innovative biological mathematical tool, Cancer Immune Control Dynamics (CICD), a clinical data driven model of systemic immunity. CICD strives to bridge biology and engineering by generating an adaptable biological model-based analysis program that incorporates multiple biomarkers. This endeavor attempts to understand and organize the individual roles of multiple measured, interacting, variably interdependent mediators (cellular and humoral) of systemic immune homeostasis (both up and down regulatory) through measurable biomarker concentrations in peripheral blood. The overall state of an individual’s immune system regardless of clinical status, is modeled as reflected in their blood samples.

Mathematical modeling concepts used in various engineering systems are the foundation of the CICD model presented herein. Physical system models simultaneously embed a multitude of complex and often ill-defined representational features including system disturbances, environment, feedback relationships, modularity, adaptability, robustness, redundancy, etc. These same ill-defined features are observed in the adaptive immune system that generate a dynamic response via a complex network of immune biomarker interactions that are heterogeneous, highly redundant and maintain homeostasis. Biological and engineering technologies appear very different but at the system level have characterizations that are mathematically equivalent and can potentially be modeled using the same principles [13–15]. To mathematically model the dynamics of the immune system, we assume that the oscillations of biomarkers’ concentrations will expose their interrelations, regardless of their disparate phenotypes and disparate functions (up vs. down regulatory). By mathematically determining which biomarkers are fluctuating together (and which are not), thereby possibly mediating each other via activation or suppression, the underlying biological complexity of the immune system is uncovered.

Mathematical modeling of biological systems, specifically the immune system has greatly increased in recent years [7]. CICD modeling utilizes the common ordinary differential equation (ODE) methods [16–18]. This approach both enables calibration against various data and is also computationally efficient making it the most widespread and flexible of models. However, its complexity grows with the addition of more equations and consequently an exponentially growing number of to-be-defined parameter values. This size problem places limits on ODE model growth potential for predictive analysis applications. To date these models are effective for well-known situations but do not fully include the ability to investigate the enormous number of possible immune interactions that can take place in the human system concurrently. CICD circumvents these limitations by applying the same ODE to each biomarker to represent all possible interactions and parameters that affect its rate of change. Patient immune parameter measurements (concentrations of peripheral blood biomarkers) populate these equations producing a mathematical representation of an individual’s immune system.

Once a system is modeled mathematically, reverse engineering strategies [19, 20] can decompose a physical system’s complex interactions to reveal otherwise hidden features, structure, and control principles. By exploiting these reverse engineering methodologies for immunology [8], the connectivity and underlying dynamics characterizations for biomarker interaction networks can potentially be exposed. CICD methodology, constructed on these principles, is able to quantify underlying biomarker relationships in the complex immune system network through an expandable, descriptive method of mathematical modeling based solely on an individual’s test period data record.

CICD results provide initial evidence that model-based systems analysis leads to patient specific clinical insights pertaining to the biology of disease (antitumor immunity) in humans. Herein we present results of our initial effort to develop an *interrogative approach* that reverse engineers in silico a model of the human immune system in patients with metastatic melanoma (and healthy volunteers) utilizing peripheral blood derived measurements (time-series) of immune function (biomarkers). This effort has adopted a data-driven approach that exploits engineering analysis methods utilized to investigate anomalous system behaviors. First verification of the computational process for this novel approach is presented in detail to validate the calculations used to identify and quantify biomarker pairings prevalent in clinical diagnoses. Next, confirmation of CICD modeling begins with the portrayal of the biological community’s accepted truths regarding homeostasis, redundancy, heterogeneity, and homogeneity.

CICD exposes the underlying causes consisting of the biomarker relationships that together affect the observed populations of the biomarkers in the blood, thereby providing an insight rich snapshot of an individual’s state of immune homeostasis. Its main objective is discovery, to help lead to improvements in the efficacy of existing immune therapies (patient selection; drug combinations) and insight into new therapeutic targets that may significantly reduce the time to discover new therapeutics capable of meaningful clinical impacts.

## Methods

### Engineering approach

**Fig. 1:**
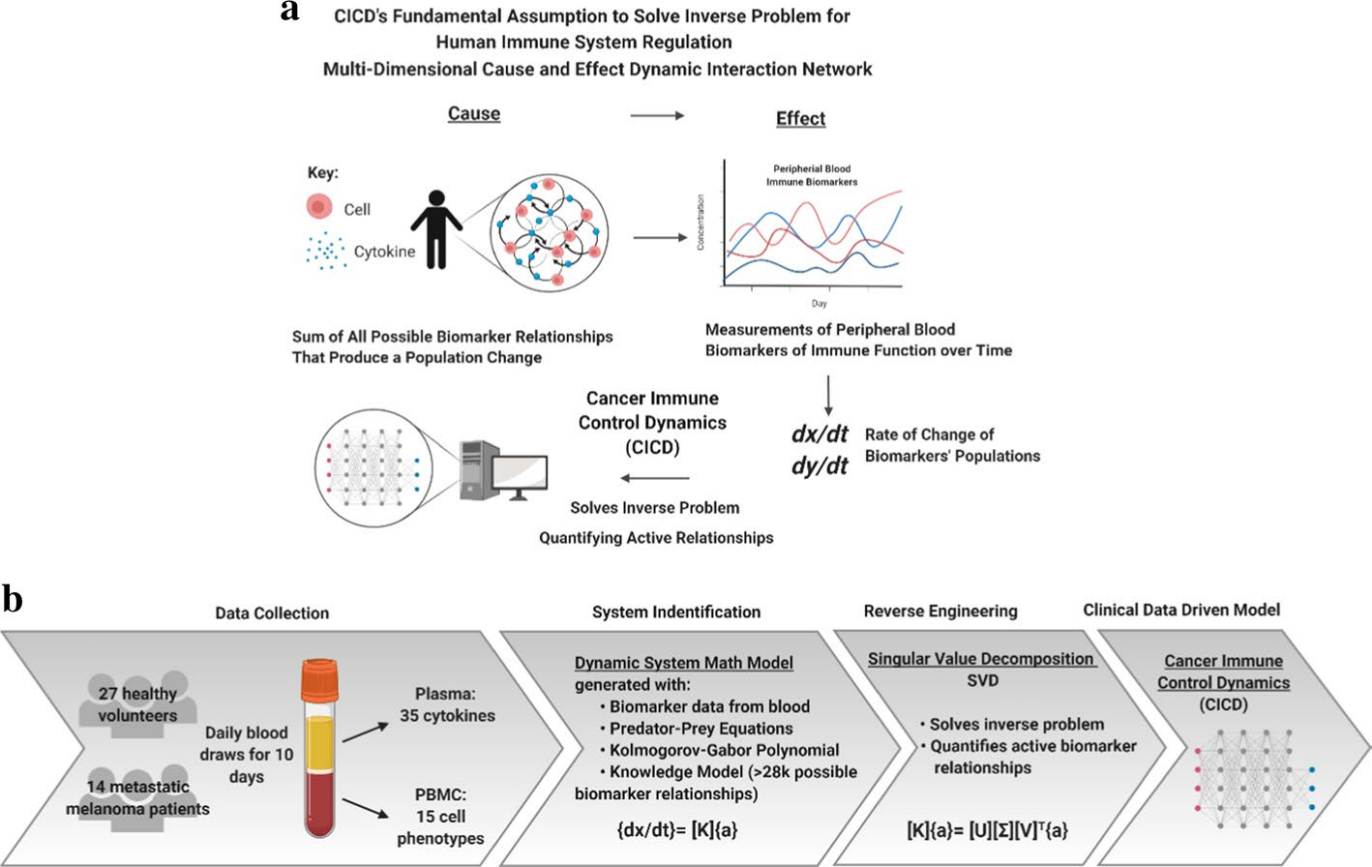
CICD engineering approach: **a** Representation of the multi-dimensional cause and effect dynamic interaction network. Multiple cell and cytokine relationships cause the effect of dynamic population changes in the measurements of the peripheral blood biomarkers of immune function over time. The CICD math model assumes that the rate of change of the effects, i.e. the observed biomarkers’ populations, is equal to the sum of all causes, i.e. the biomarker relationships that produce a change in a biomarker population. With this time series data CICD is able to solve the inverse problem to quantify specific active relationships that manifest in the oscillations of biomarker concentrations. **b** Foundational architecture for CICD analysis process. Daily blood draws were performed to obtained daily measurements of both cell and cytokine immune biomarkers. CICD’s system identification process then creates a system characterization matrix to provide a dynamic system math model with biomarker data, predator–prey equations, a truncated Kolmogorov–Gabor polynomial and thousands of possible biomarker relationships. Reverse engineering analysis uses a Singular Value Decomposition algorithm (SVD) to solve the inverse problem and identify a solution profile of the active biomarker relationships. With CICD modeling capabilities, the complexity of the immune system is mathematically quantified through thousands of possible interactions between multiple biomarkers

CICD’s modeling methodology belongs to a specialized sub-discipline known as inverse problems, important mathematical problems in science and mathematics because they provide information about parameters that are not directly observed. In essence, an inverse problem in science consists of the process of calculating from a set of observations the causal factors that produced them. CICD’s fundamental assumption (Fig. 1a) is that a multi-dimensional cause and effect dynamic biomarker interaction network occurs between biomarker populations in the immune system. CICD uses the observed measurable output populations to mathematically calculate the activity of the underlying input interactions that are potential signals utilized to control the immune system. CICD takes advantage of the oscillatory nature of the changes in biomarker concentrations over time in order to resolve inter-parametric relationships that result in the maintenance of systemic immune homeostasis in cancer versus healthy.

CICD utilizes principles of control theory to examine the internal dynamics of the human immune system. As in mechanical systems, a sensor provides continuous feedback information to a controller to induce a response to maintain the stability (homeostasis) of the system. Prior to distinguishing biological embedded sensors and controllers, a model first needs to be developed through system identification which builds a mathematical model of the dynamical system from measured data. With a viable mathematical model, additional engineering principles and algorithms are applied to reveal the underlying causes or signals that provide information to the system [9]. CICD’s innovation is that it provides a quantitative math model representation for the dynamic interaction network of the immune system based on serially collected peripheral blood measurements. Figure 1b provides the foundational architecture of CICD’s analysis process. The system identification process builds the math model by (1) utilizing the serially collected peripheral blood samples (biomarker data) as measured data for patient biological status, (2) representing the biomarker population dynamics as mathematical relationships between measured data expressed as non-linear ordinary differential Lotka–Volterra predator–prey equations, and (3) creating a biomarker interaction network, through a matrix generalization of these equations using a truncated Kolmogorov–Gabor polynomial to include all possible biomarker relationships as specified in the Knowledge Model. These components generate predator–prey equations for each biomarker studied that are combined into a system characterization matrix to provide a dynamic behavior model. This matrix is the foundation for the reverse engineering analysis. This math model is reverse engineered using methods of linear matrix computation [11], specifically Singular Value Decomposition (SVD) to approximate all biologically possible nonlinear cause and effect coupling mechanisms. The composite resultant data exposes the most active biomarker relationships or causes that associate with observed effects as measured in the blood data. This unique modal analysis solution process creates a database of information that can be mined for characterizing patient and multi-patient cause and effect network interaction dynamics.

The development of CICD has evolved through collaboration between medical and engineering disciplines using extensive control theory experience in the computational modeling of ill-conditioned systems. The associated vision for the analysis framework for how CICD’s model-based information should be formulated, collected and presented has been influenced by multiple contributing sources [10, 13, 16, 21–25].

### CICD patient biological status

The data used consists of 35 cytokines (plasma concentrations): EGF, EOTAXIN, FGF-2, FLT-3L, FRACTALKINE, G-CSF, GM-CSF, IFNa2, IFNg, IL-10, IL-12p40, IL-12p70, IL-13, IL-15, IL-17A, IL-1a, IL-1b, IL-1ra, IL-2, IL-3, IL-4, IL-5, IL-6, IL-7, IL-8, IL-9, IP-10, MCP-1, MCP-3, MIP-1a, MIP-1b, TGFa, TNFa, TNFb, VEGF and 15 cell phenotypes (relative concentrations among peripheral blood mononuclear cells): CD11c+, CD11c/CD14+, CD11c/CD86+, CD11c/HLA-DR+, CD123/HLA-DR+, CD14/CD197+, CD16/CD56+, CD3+, CD3/CD4+, CD3/CD62L+, CD3/CD69+, CD3/CD8+, CD4/CD294+, CD4/TIM3+, and CD56+. Blood samples are obtained at approximately the same time of day Monday thru Friday over a 2-week period in 14 patients with stage IV metastatic melanoma, not on active therapy as well as in 27 healthy volunteers. Cells and cytokines are collectively referred to as biomarkers. Computational analysis for system oscillatory dynamics builds upon the implicit assumption that data sampling is a continuous dynamic process for which characterization models are derived from available data records. Herein linear interpolation defines the biomarker rate of change, slope, as constant between blood draws providing an initial, simple, numerical approximate of the entire test period response. CICD analysis is valid at any instant between blood draws. Six equally timed sampling points are chosen between each draw to approximate dynamic biomarker actions executed between blood draw instances. The methods used to obtain the clinical data is found in Additional file 1.

### CICD model of biomarker population dynamics

A mathematical expression that can model biomarkers’ fluctuations over time in relation to each other’s growth and decay is needed to determine the prominent relationships. The Lotka–Volterra predator–prey equations are first-order nonlinear differential equations used to describe the dynamics of biological systems in which two species interact. To “translate” these mathematical equations into immune biology, each cell and cytokine is viewed as an individual species. The predator–prey approach merely provides a simple mathematical framework to organize the competing influences of the numerous immune-activating and immune-suppressing sets of signals involving both cellular and cytokine biomarkers. CICD generalizes these established constitutive relationship equations to include multiple biomarkers to model their interacting populations. The state variables of immunology are biomarker population size observed in blood (measured concentrations). This modeling approach provides the ability to capture concurrently oscillatory, exponential growth and decay behaviors between interacting biomarker populations as they are continuously being used and replaced (concentrations going down and up, respectively). Each biomarker’s population change is expressed via an equation, which CICD then uses to build a large, generalized, biomarker interaction matrix model.

### CICD model of biomarker interaction network

**Fig. 2:**
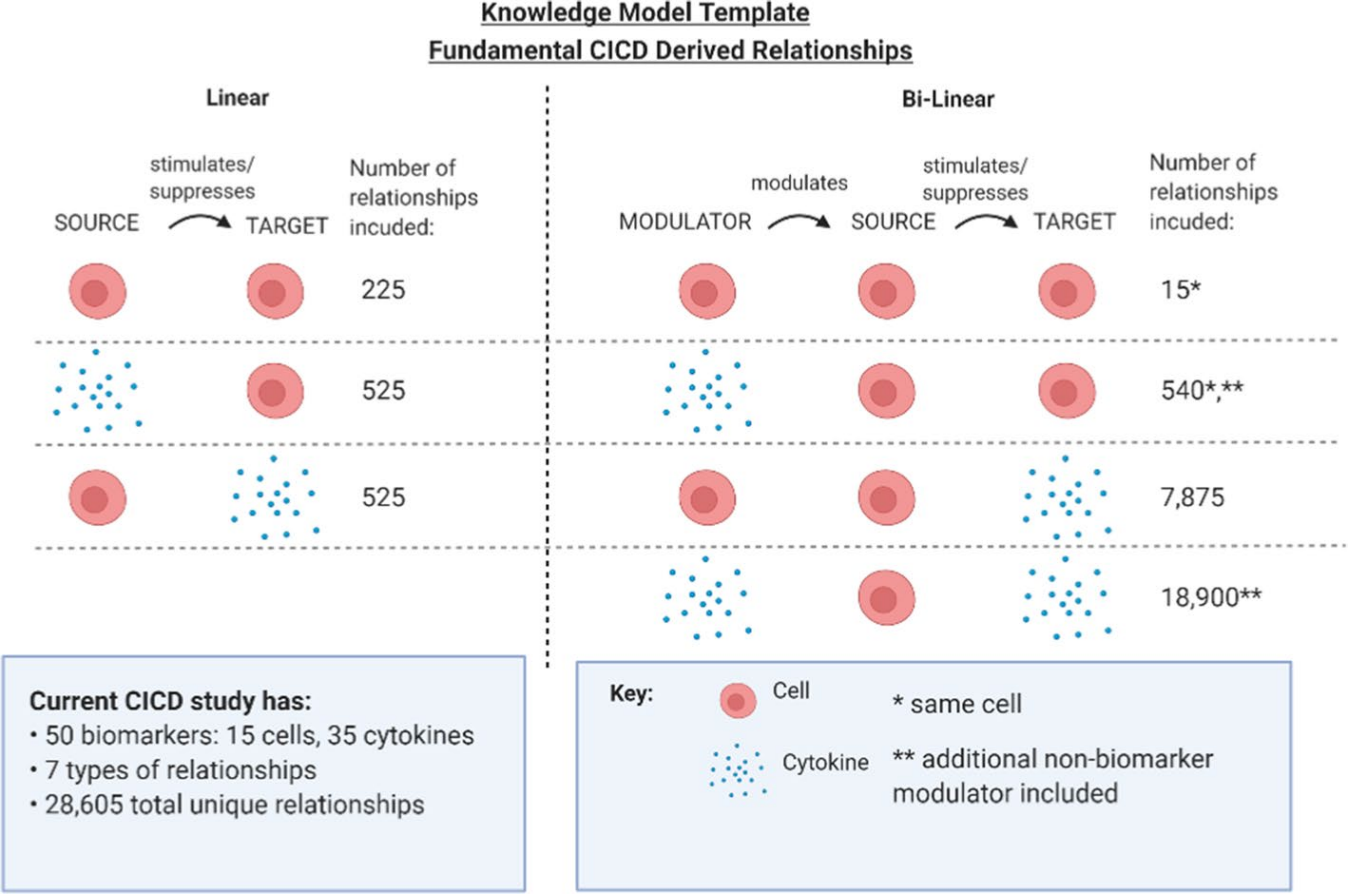
Knowledge model template of the fundamental CICD derived relationships. The fundamental CICD derived relationship is a Modulator biomarker modulates a Source biomarker that stimulates or suppresses a Target biomarker. Modulator is a biomarker that modulates a Source biomarker. Source is a biomarker that stimulates or suppresses a Target biomarker. Target is a biomarker that is stimulated or suppressed by either a modulated Source biomarker or a non-modulated Source biomarker. The Knowledge Model for the current study is a template of 7 relationship criteria to reduce number of relationships analyzed to a total of 28,605 unique relationships. Each type of CICD derived relationships is shown with the total number of combinations included in the current Knowledge Model. For each relationship, a cell is required to be either a Target or a Source component. Bi-linear relationships are those in which the Modulator affects the Source, then this Source affects the Target. Bi-linear relationships with more than one cellular component requires it is the same cell unless it acts as a Modulator for a cytokine Target; that is, autocrine signaling is included and for simplicity purposes paracrine signaling is currently excluded. Linear relationships are a non-modulated Source biomarker acting directly on a Target biomarker. *Relationship requires the same cell used for each cellular component. **An additional modulator has been included to model possible non-biomarker modulations

The fundamental CICD derived relationship consists of three components “Target”, “Source”, and “Modulator”, modeled as the population of the Target biomarker is stimulated or suppressed by the population of the Source biomarker that is either modulated by the population of the Modulator biomarker (bi-linear) or is not modulated (linear) (Fig. 2). This relational concept provides a means to create a profile of the dynamic immune system by defining thousands of sets of potential immune signals (up and down regulatory). A major design goal for CICD is to identify and quantify only the biologically possible underlying networks of connectivity in the human immune system. To ensure the broad applicability of CICD’s analysis methodology, a systems-engineering approach is adopted where theoretically all combinations can be included while allowing the user an ability to reduce or expand the characterization model according to the needs of the study. Through quantification of the observed behaviors of these possible causes, CICD creates a descriptive mathematical model of what has occurred in contrast to predicative models that hypothesize what will occur.

CICD uses a template, referred to herein as the Knowledge Model, to generate a comprehensive list of biomarker relationships that may cause a change in a biomarker population. In order to maintain biological integrity, a template is incorporated to identify specific biologically possible combinations for the analysis according to the biomarker’s type (Fig. 2). The Knowledge Model template states which group, cells or cytokines, can populate each component. With these criteria, currently 7 types of relationships, the 35 cytokines and 15 cells studied generate 28,605 unique biomarker relationships. This allows the application of system identification concepts to generalize the predator–prey equations to a mathematical model that approximates specific biologically possible relationships according to the study’s needs and is consistent with well-established biological knowledge. The generalization provides the framework to include all possible causes, i.e. biomarker relationships that affect the biomarker population rate of change.

Once all relationships to be included are determined, the Kolmogorov–Gabor polynomial (Eq. 1) models all actions that drive cell-cytokine interaction network dynamics. It provides a framework to express each possible population change cause as an atomic element in the equation, as a linear (non-modulated source) or bi-linear (modulated source) product of state variables, each with an associated unknown parameter. Based on CICD’s multi-dimensional cause and effect assumption, the rate of change of a Target biomarker is expressed as being equal to the algebraic sum of each atomic element for every CICD derived relationship as dictated by the Knowledge Model.

The generic format of the Kolmogorov–Gabor polynomial is:

1 $$Y\left( {x_{1} , \ldots , x_{n} } \right) = a_{0} + \mathop \sum \limits_{i = 1}^{n} a_{i} x_{i} + \mathop \sum \limits_{i = 1}^{n} \mathop \sum \limits_{j = i}^{n} a_{ij} x_{i} x_{j } + \cdots$$

Herein, each variable of state $x_{1} , \ldots , x_{n}$ represents a biomarker population, (Sources and Modulators), the *a*’s are the unknowns of the atomic elements of population change, and $Y\left( {x_{1} , \ldots , x_{n} } \right)$ is the resultant of all possible relationships as dictated by the Knowledge Model that impact one system biomarker (Target). The unknown (*a*’s) incorporate all known and unknown underlying mediating parameters. CICD truncates the polynomial (Eq. 1) at the bi-linear term to maintain SVD solution accuracy consistent with the square root of machine precision as well as limit problem size. The polynomial models all biomarkers relationships by providing a framework to express the set of Target equations (herein, 50) in linear matrix form (Eq. 2). This matrix provides the characterization of the immune regulatory system interactions at any time instant during the test period.

### CICD model of reverse engineering

The system dynamics analysis relationship is expressed in linear matrix form (Eq. 2) as:

2 $$\left\{ {\frac{dx}{{dt}}} \right\} = \left[ K \right]\left\{ a \right\}$$

3 $$\left[ K \right]\left\{ a \right\} = \left[ U \right]\left[ {\Sigma } \right]\left[ V \right]^{T} \left\{ a \right\}$$

Herein $\left\{ {\left. {\frac{dx}{{dt}}} \right\}} \right.$ is the short (50 × 1) vector of biomarker state variables, $\left\{ a \right\}$ is the long (28,605 × 1) vector of all linear and bi-linear relationship unknowns and $\left[ K \right]$ is the long thin (50 × 28,605) rectangular matrix of Kolmogorov polynomial state variable linear and bi-linear scalar products. For all atomic element constitutive relationships implicit in equation (Eq. 1), the CICD characterization matrix $\left[ K \right]$ is completely quantified by biomarker values. The participant’s biomarker data populates each of the Knowledge Model’s 28,605 relationships for every analysis time point, currently 48 for 10 days of serial blood collections. This step of data preparation prior to the SVD calculation generates 48 uniquely valued matrixes each containing 28,605 non–zero data points for every participant. This non-typical predator–prey equation format creates a linear matrix algebraic relationship between the time instant biomarker change vector $\left\{ {\left. {\frac{dx}{{dt}}} \right\}} \right.$ and all Kolmogorov Polynomial unknowns. The net result is a SVD-friendly re-formulation of the non-linear predator–prey ODE dynamics problem into an identical linear ODE problem that has a well-defined matrix $\left[ K \right]$ of time varying coefficients and a computable vector of unknowns, i.e. $\left\{ a \right\}$ for every time instant for which $\left\{ {\left. {\frac{dx}{{dt}}} \right\}} \right.$ is defined. By making the plausible linear interpolation hypothesis for all biomarker response signals inserted between measured data points, a well-defined constant value for $\left\{ {\left. {\frac{dx}{{dt}}} \right\}} \right.$ is obtained. The second equality sign introduces the SVD (Eq. 3) relationship that equates matrix $\left[ K \right]$ to the matrix triple product of Left Singular Vectors (LSV’s) in $\left[ U \right]$, singular values in $\left[ {\Sigma } \right]$ and Right Singular Vectors (RSV’s) in $\left[ V \right]^{T}$. These orthogonal vectors decompose the time instant set of fully coupled biomarker system equations into an equally sized set of fully decoupled generalized coordinate system equations. Singular values are scale factors associated with all ortho-normalized LSV’s and RSV’s.

From the mathematical viewpoint, CICD fundamental Eqs. (2) and (3) directly equates the blood sample input data to the CICD results output. Input data totally defines contents of $\left\{ {\left. {\frac{dx}{{dt}}} \right\}} \right.$ and $\left[ K \right]$. Computed results totally define the contents of $\left\{ a \right.\}$, $\left[ U \right], \left[ \Sigma \right]$ and $\left[ V \right]^{T}$. CICD analysis ensures that the resultant data is accurate to machine precision by having a quality check incorporated into the program. The resultant data, the computed values for $\left\{ a \right.\}$, $\left[ U \right]$, $\left[ \Sigma \right]$ and $\left[ V \right]^{T}$ is used to back compute the input data to ensure both the original input data and recalculated input data are within expected machine precision bounds. This validation ensures the integrity and numerical stability of the computed results.

An SVD algorithm variant specialized to work with the very long thin (50 × 28,605) rectangular matrices is utilized by CICD to circumvent well-known numerical computation problems associated with several nearly equal singular values [26]. Conceptually, the SVD iterative solution process creates a sequential product of similarity transformations that is stopped before an ill-conditioned computing step is executed. The net result is a minimum norm, least effort, solution that has machine solution accuracy and a LSV matrix $\left[ U \right]$ that is not unit diagonal. This specialized SVD decomposition of the system characterization matrix $\left[ K \right]$ is key to CICD’s ability to reverse engineer a patient’s test period record to quantify all active causes that result in a biomarker population change. CICD analysis uncovers and quantifies the active biomarker relationships specific to an individual’s immune network through the interaction strength values for all LSV’s and RSV’s elements.

### CICD model of biomarker cause and effect interaction network

**Fig. 3:**
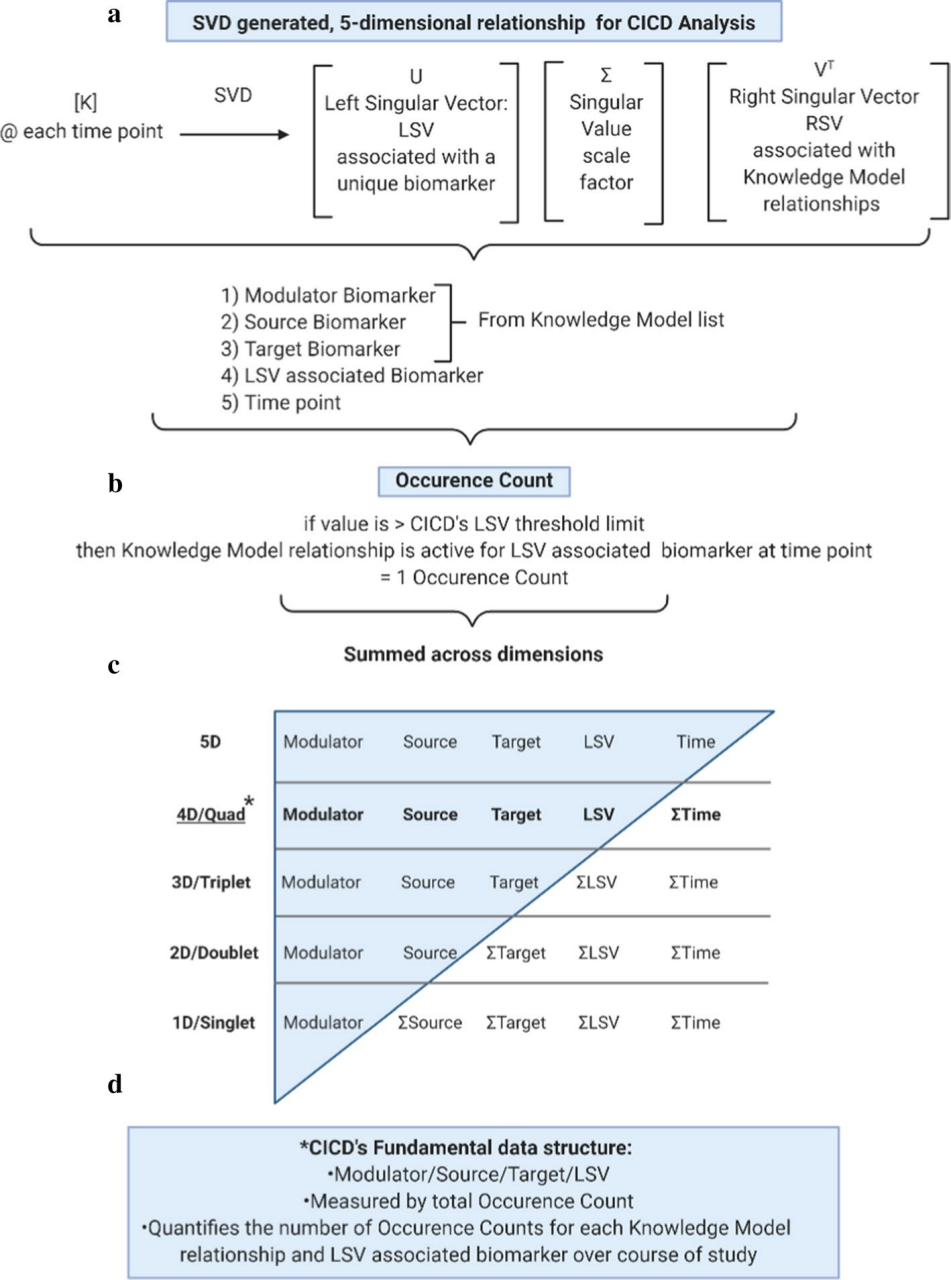
CICD’s flow chart for data processing and interpretation. **a** SVD generated 5-dimensional relationship. SVD calculations adds two additional dimensions to the Knowledge Model relationships, an LSV associated biomarker and a time point, **b** Occurrence Count. A Knowledge Model relationship is active for a LSV associated biomarker at a single time point if its value is above the determined LSV threshold. This is counted as one Occurrence Count. **c** Σ Summation series of CICD result data. Σ Summations across like components generates multiple test period resultant views of the CICD data. The initial CICD summation involves the time instant dimension. The active 5-D relationships are summed across time to produce a unique Quad relationship containing its Modulator, Source, Target and LSV component. Next, the clear patterns of Quad activity enable the ability to sum the number of active occurrences with the same biomarker components to produce what is referred to herein as Triplets, Doublets, and Singlets. The LSV components are combined to create the 3-dimensional Triplet view. The next summations can be performed across any of the remaining components, Target, Source or Modulator to create three, 2-dimensional Doublet views, Modulator-Source (shown), Source-Target, Target-Modulator. Singlet views is the sum of all Occurrence Counts for a biomarker acting as a Modulator (shown), Source, Target, or LSV component. **d** CICD’s Fundamental data structure, Quad relationship. The SVD generated LSV quantifies the number of active occurrences for each Knowledge Model relationship over the course of the study and is the basis for analysis presented

Once SVD calculates the resultant data structures, they must be interpreted to understand the biological interactions relative to the measured input data. Figure 3 provides a flow chart on how the large relational data structure is processed to interpret and extract biologically relevant insight.

CICD analysis decomposes a $\left[ K \right]$ matrix by SVD at every analysis time point during the test period so that each LSV column of $\left[ U \right]$ has an associated RSV row of $\left[ V \right]^{T}$ that contains all possible relationships as defined by the Knowledge Model (Fig. 3a). This results in two additional dimensions to the current three-dimensional CICD derived relationship. Therefore, at each time point, the SVD calculation generates a CICD relationship with 5 dimensional components and an associated value: (1) test period time instant, (2) LSV column, (3) Target biomarker, (4) Source biomarker, and (5) Modulator biomarker.

The SVD variant computes 50 LSVs corresponding directly to the number of biomarkers analyzed. To connect the math of Eq. (3) to biology it is necessary to label each specific LSV column of $\left[ U \right]$ and RSV row of $\left[ V \right]^{T}$. This effort is non-trivial. At every analysis time instant, a name-tagging algorithm associates a unique biomarker name to each LSV according to its dominant biomarker component. This associates each LSV natural mode of behavior with a specific biomarker. Ambiguous cases exist per LSV matrix $\left[ U \right]$ but are relatively few in which additional steps are utilized. This labeling method ensures that every LSV is associated with a unique biomarker nametag according its behavioral activity.

CICD methodology includes a small set of response threshold rules to reflect the biology response expectation that LSV solutions will have a clearly identified dominator component and a modest number of supporter components, while removing very small valued interactions. For the resultant data analysis, all LSV components with values above the threshold are considered active. Therefore, for each 5-dimensional relationship if its value is greater than the threshold limit it is assumed active and counted as a single Occurrence Count (Fig. 3b).

Each CICD calculated unknown value is assumed to represent all parameters acting on its specific relationship. Herein we removed those relationships with an unknown value of zero, per the original equation in which each term is the product of the unknown and the state variables representing the total causal effect of the specific biomarker relationship. If this effect is equal to zero, then it is assumed not active and is not included in the current investigation.

A key data processing step of the result data is a series of (Σ) summations to expose the highly active biomarker networks. Figure 3c illustrates the multiple views of the data that are generated by combining CICD relationships with like components. Each level provides insight into an individual’s immune state via several resultant test period relationships that can be explored for possible clinical insight.

CICD via SVD, computes 50 Singular Values with an associated set of Left and Right Singular Vectors (LSV, RSV). Each LSV has 50 components, one for each biomarker. Each RSV has 28,605 components, one for each possible biomarker relationship defined by the Knowledge Model in the current study. For modal analysis, this generates 50 × 28,605 unique 4-dimensional relationships, each with a unique Knowledge Model relationship and an associated LSV across the time period creating CICD’s fundamental data structure, the Quad relationship (Fig. 3d). Therefore, the Quad relationship contains a unique Modulator, Source, Target, and LSV combination with the number of Occurrence Counts over the test period.

The CICD analysis establishes a new unique measurement, Occurrence Count that quantifies the influence of these biomarkers relationships or signals in an individual’s immune system. The total Occurrence Count is the number of times each specific Quad is active during the test period. The maximum Occurrence Count for each Quad corresponds directly to the number of blood draws and number of time instances analyzed, herein 48 for the 10 days of serial peripheral blood sample collections. To aid current patient comparisons as well as potential future studies (reducing the number of days for serial peripheral blood collections), each Quad’s Occurrence Count is divided by the maximum count as dictated by the number of time points then multiplied by 100. This normalization provides a consistent maximum value independent of study length as well as a representation of the percentage of activity for the Quad across the study period. Therefore, active Quads have a range of Occurrence Counts from 0 to 100. The CICD resultant data analyzed herein is the normalized Occurrence Count value of each unique Quad and is the basis for all result figures presented. CICD analysis presented herein originates with the Quads that are combined by LSV and Targets to obtain the Source-Modulator Doublets in which high variation was observed across individuals. The principal results illustrate the variability of the Doublet Occurrence Counts across patient and clinical groups.

## Results

### Patient peripheral blood derived serial biomarker data

**Fig. 4:**
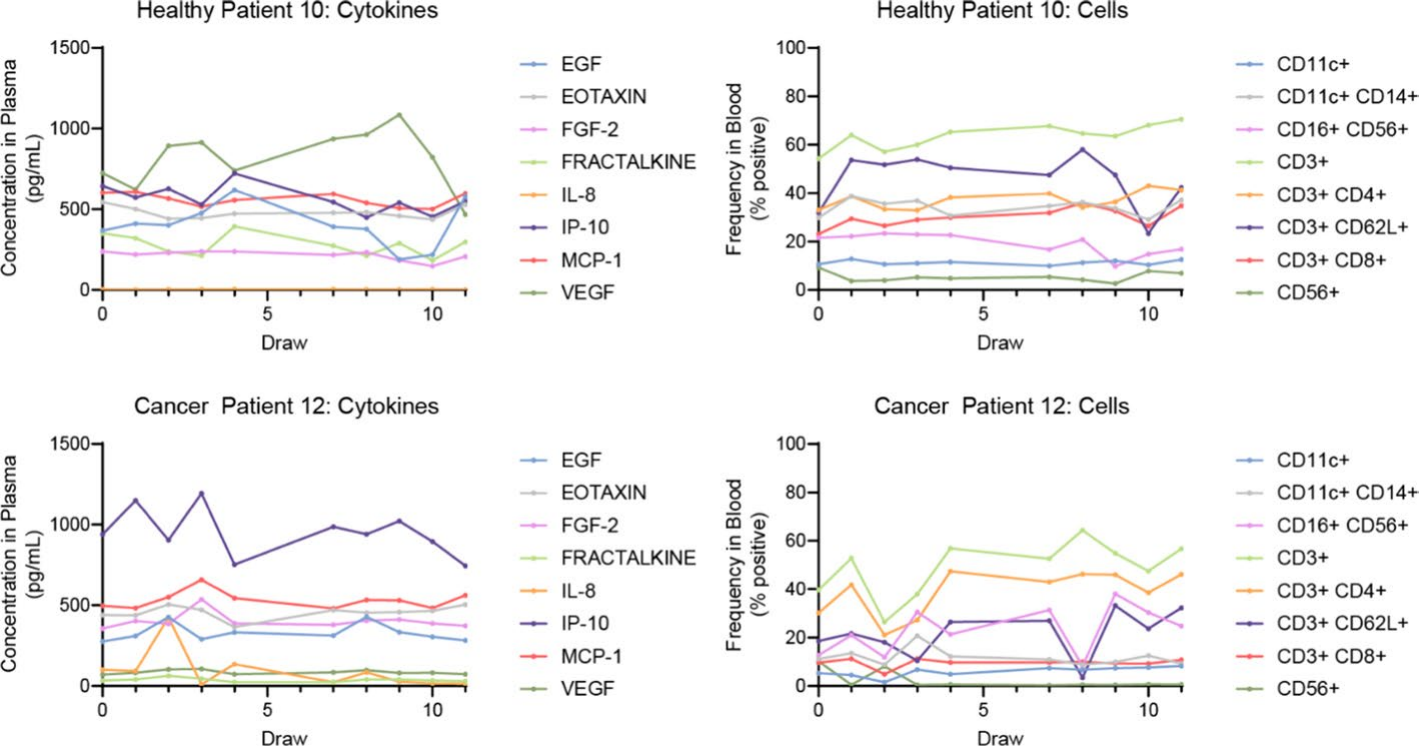
Patient peripheral blood derived serial biomarker data. A data sample of 8 cytokines and 8 cells for one healthy volunteer (#10) and one cancer patient (#12A) across a study time period demonstrates the highly dynamic and fluctuating nature of the immune system. Values are the measured biomarker concentrations. Biomarker values are connected via linear interpolation, which approximates the oscillatory behaviors of the biomarkers over time, thus providing the computational analysis with a first order approximant model for all test period response data

A sample of the peripheral blood data is presented in Fig. 4. 50 biomarkers are analyzed for both cancer patients and healthy volunteers across the 2-week test period (consecutive weekdays only). Biomarker values vary both across time and between individuals. The highly dynamic and fluctuating nature of the immune system is demonstrated through a sample of the CICD biomarker input data for an individual cancer patient and a healthy donor. These graphs illustrate the overall general similarities and the deviations found between the study groups. It is these variations that CICD seek to quantify via underlying unobservable interactions that manifest in the observed and measurable concentrations. Each participant’s graphs of cell and cytokines concentrations as shown in Fig. 4 are found in Additional file 2.

### CICD data analysis

A verification of methodology is presented through the findings that only 133,000 Quads out of a possible 1.4 million are non-zero across all participants and only several thousand for an individual. The CICD final results consist of a list of active Quad relationships and their normalized Occurrence Count for each patient.

**Fig. 5:**
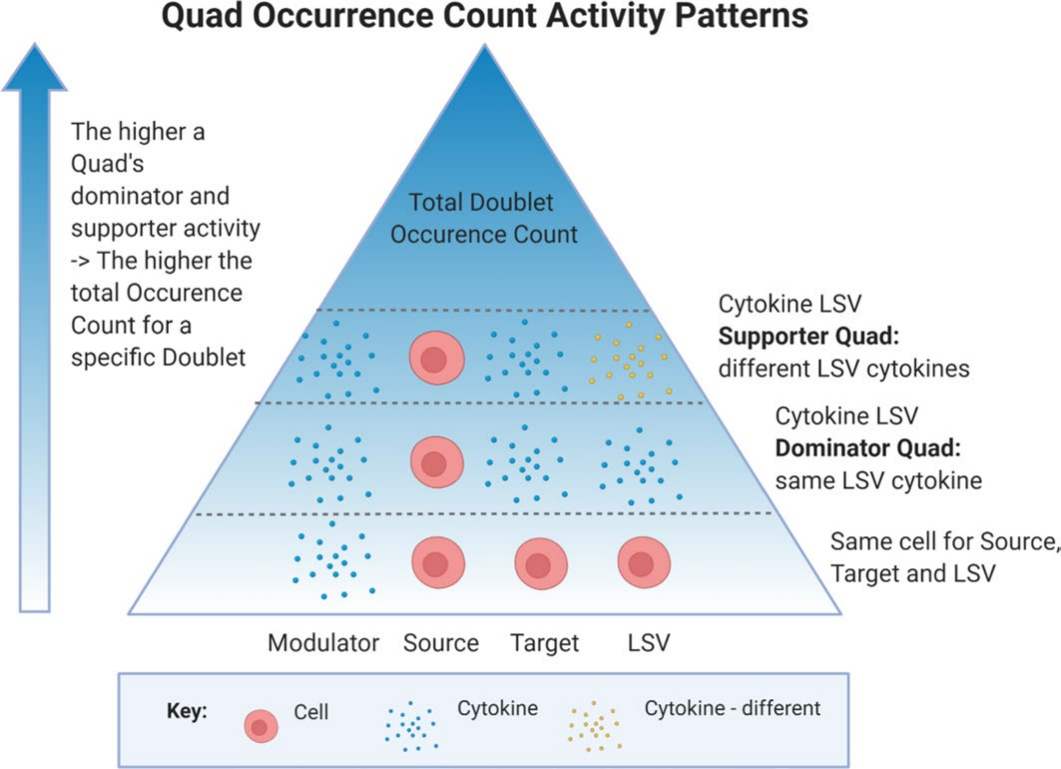
Quad Occurrence Count activity patterns. Observed Occurrence Counts for the bi-linear Quads across all participants present in similar cumulative pattern as shown. If a Doublet or Triplet has an Occurrence Count, first the Quad with the same cell in the Source, Target, and LSV must be active. Second, a Dominator Quad (same cytokine in the Target and LSV) Occurrence Count is observed. Finally, if both types of bi-linear Quads are active then Supporter Quads (different cytokine in the Target and LSV) Occurrence Counts are observed. Therefore, the higher the Doublet Occurrence Count the more activity is found in the Dominator and Supporter Quads

The total Occurrence Count for each Quad, Triplet, Doublet and Singlet is explored and compared between patients and patient groups to reveal active biomarker interactions. Table 1 provides a small sample of the thousands of active Quads for a cancer patient and a healthy donor, furthermore it illustrates the summation process as depicted in Fig. 3c for Triplets (Modulator, Source, and Target) and Doublets (Modulator and Source). Due to the large dataset only one Target, IL-8 for the Modulator-Source Doublet, IP-10/CD3 + is provided herein, but it has been observed throughout datasets that a consistent pattern of activity is repeated for Targets/LSVs in an active Doublet. Three levels of Target/LSV activity have been continuously observed throughout all Quad datasets (Fig. 5). First, if the Doublet is active it will have at a minimum an Occurrence Count for the Quad with the same cell in the Source, Target, and LSV (Quad: IP-10/CD3+/CD3+/CD3 +). The Source cell stimulates/suppresses itself. The next level of observed activity is in the Dominator LSV, the Quad with the same cytokine in the Target and LSV (Quad: IP-10/CD3+/IL-8/IL-8). The redundancy of the system is illustrated by the final level of activity, the Supporter LSVs, the Quads with a different LSV cytokine from the Target (Quad: IP-10/CD3+/IL-8/non-IL-8 Cytokines). These additional Occurrence Counts are viewed as supporting activity for the Triplet, increasing the effects on the Target cytokine. It has been observed that if a specific Doublet is found active in a specific patient, the Occurrence Counts for the cell Target, Dominator LSV and Supporter LSV activity presents in a similar pattern. The supporting activity varies across the LSVs but is only observed if both the cell Target and the Dominator LSV Quad are active for the Triplet providing the justification for the summation process. This cumulative pattern of Occurrence Counts manifests in the total Modulator-Source Doublet Occurrence Count, the higher the sum value, the higher the level of supporting LSVs across all Targets, and therefore the higher its overall effects on all biomarkers Targets. Much of the CICD result data reflects observations seen in the biomarker data that cannot be currently quantified. For the two study participants provided in the paper, the IL-8 input data (blood biomarker concentrations) in the cancer patient is higher and appears to be oscillating in the same pattern as IP-10 compared to the presented healthy volunteer (Fig. 4). For the same two study participants, both similarities and differences for their Occurrence Counts are observed for these biomarkers in the specific sample of Quads provided in Table 1. Here IP-10 is modulating CD3 + which is targeting CD3 + and IL-8 across several biomarkers’ LSV. The cancer patient in this example has a much higher level of activity for this IP-10/CD3+/IL-8 Triplet and IP-10/CD3 + Doublet generated from various supporting biomarker LSVs as compared to this specific healthy volunteer. Therefore, according to CICD analysis the relationship IP-10/CD3 + is affecting multiple cell and cytokine biomarkers more intensely in this cancer patient as compared to the healthy volunteer. Also, IL-8 is acting as a non-modulator source on several cellular targets but only for the cancer patient. These specific Knowledge Model relationships are now quantified by CICD result data. The Triplets and Doublets compared here are only a few of the thousands of observable comparisons found between the study participants and clinical groups (cancer vs. healthy) with CICD, illustrating that variations observed in the biomarker blood data can have many underlying causes that can be mathematically uncovered. This summation process is repeated for all Quads and additional software has been developed specifically tailored to explore CICD data both within and across patients.

**Table 1:** CICD data analysis for cancer patient 12A and healthy volunteer 10

BI-linear, modulated relationships				Cancer patient 12A		Healthy volunteer 10	
Quad: Modulator, Source, Target, LSV							
Triplet: Modulator, Source, Target							
Doublet: Modulator, Source							
Modulator	Source	Target	LSV	OCC. Count	Sum	OCC. count	Sum
IP-10	CD3+	CD3+	CD3+	100.00		100.00	
**IP-10**	**CD3+**	**CD3+**			**Triplet: 100**		**Triplet: 100**
IP-10^a^	CD3+^a^	IL-8^a^	IL-8^a^	93.75		89.58	
IP-10	CD3+	IL-8	EOTAXIN	35.42		2.08	
IP-10	CD3+	IL-8	IP-10	29.17		2.08	
IP-10	CD3+	IL-8	MCP-1	29.17		6.25	
IP-10	CD3+	IL-8	VEGF	25.00		6.25	
IP-10	CD3+	IL-8	TGFA	20.83		16.67	
IP-10	CD3+	IL-8	IL-1A	20.83		8.33	
IP-10	CD3+	IL-8	EGF	18.75		4.17	
IP-10	CD3+	IL-8	IL-12P40	14.58		2.08	
IP-10	CD3+	IL-8	FGF-2	14.58		2.08	
IP-10	CD3+	IL-8	FRACTALKINE	14.58		4.17	
IP-10	CD3+	IL-8	G-CSF	12.50		0.00	
IP-10	CD3+	IL-8	IL-12P70	12.50		0.00	
IP-10	CD3+	IL-8	IL-1RA	12.50		4.17	
IP-10	CD3+	IL-8	IL-3	12.50		2.08	
IP-10	CD3+	IL-8	MCP-3	12.50		2.08	
IP-10	CD3+	IL-8	TNFB	12.50		2.08	
IP-10	CD3+	IL-8	IFN-G	10.42		0.00	
IP-10	CD3+	IL-8	IL-2	10.42		0.00	
IP-10	CD3+	IL-8	MIP-1A	10.42		0.00	
IP-10	CD3+	IL-8	GM-CSF	8.33		0.00	
IP-10	CD3+	IL-8	IFN-2A	8.33		2.08	
IP-10	CD3+	IL-8	IL-10	6.25		0.00	
IP-10	CD3+	IL-8	IL-13	6.25		4.17	
IP-10	CD3+	IL-8	IL-17A	6.25		0.00	
IP-10	CD3+	IL-8	IL-1B	6.25		4.17	
IP-10	CD3+	IL-8	IL-4	6.25		4.17	
IP-10	CD3+	IL-8	IL-9	6.25		4.17	
IP-10	CD3+	IL-8	MIP-1B	6.25		0.00	
IP-10	CD3+	IL-8	TNFA	6.25		2.08	
IP-10	CD3+	IL-8	FLT-3L	4.17		0.00	
IP-10	CD3+	IL-8	IL-7	4.17		2.08	
IP-10	CD3+	IL-8	IL-15	2.08		2.08	
IP-10	CD3+	IL-8	IL-6	2.08		4.17	
IP-10	CD3+	IL-8	IL-5	0.00		0.00	
**IP-10**	**CD3+**	**IL-8**		**502.08**	**Triplet: 502.08**	**183.33**	**Triplet: 183.33**
**IP-10**	**CD3+**				**Doublet: 11,839.58**^b^		**Doublet: 5210.42**^b^

Linear, non-modulated relationships				Cancer patient 12A		Healthy volunteer 10	
Quad: Modulator, Source, Target, LSV							
Triplet: Modulator, Source, Target							
Doublet: Modulator, Source							
Modulator	Source	Target	LSV	OCC. Count	Sum	OCC. Count	Sum
n.a	IL-8	CD3/CD69+	CD3/CD69+	18.75	**Triplet: 18.75**	0.00	**Triplet: 0**
n.a	IL-8	CD11c/CD86+	CD11c/CD86+	10.42	**Triplet: 10.42**	0.00	**Triplet: 0**
n.a	IL-8	CD11c/HLA-DR+	CD11c/HLA-DR+	10.42	**Triplet: 10.42**	0.00	**Triplet: 0**
n.a	IL-8	CD11c+	CD11c+	8.33	**Triplet: 8.33**	0.00	**Triplet: 0**
n.a	IL-8	CD4/CD294+	CD4/CD294+	6.25	**Triplet: 6.25**	0.00	**Triplet: 0**
n.a	IL-8	CD4/TIM3+	CD4/TIM3+	6.25	**Triplet: 6.25**	0.00	**Triplet: 0**
n.a	IL-8	CD56+	CD56+	2.08	**Triplet: 2.08**	0.00	**Triplet: 0**
**n.a**	**IL-8**			**83.33**	**Doublet: 83.33**	**0.00**	**Doublet: 0**

43 of the 10,869 active Quad’s for the cancer patient and 25 of the 10,757 active Quad’s for the healthy volunteer are presented. Every row represents one unique active Quad containing the four components (LSV, Target, Source, and Modulator) and a value for its Occurrence Count. Examples of both bi-linear, modulated relationships (top section) and linear non-modulated relationships (bottom) are shown. The Triplets have the same Target, Source, and Modulator and its value is the sum of all its LSVs Occurrence Counts. The Doublets have the same Source and Modulator, combining all Target and LSV biomarkers. The value is the sum of all Occurrence Counts that have this common Doublet within its Quad.

^a^Dominator LSV, Target and LSV are the same cytokine in Quad

^b^Actual Doublet Value is higher due to additional Quads not shown

### CICD modeling of biologic interactions

**Fig. 6:**
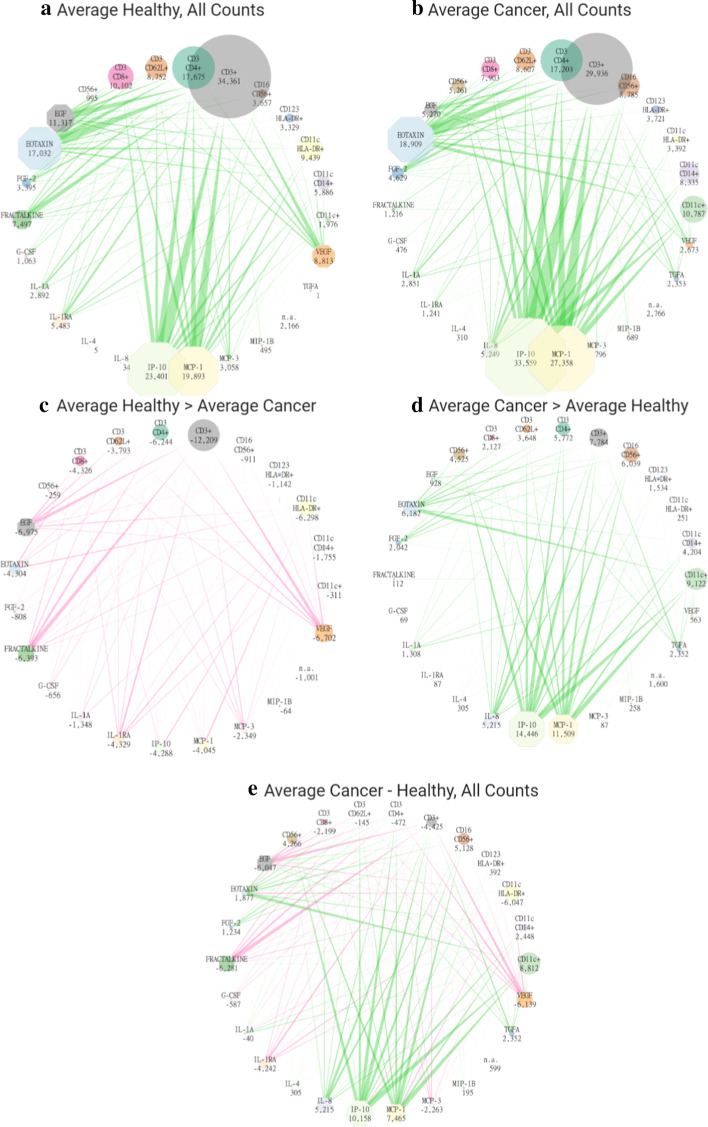
Average Healthy versus Average Cancer flow diagrams. Each flow diagram begins with a Quad dataset that is reduced to Doublets (edges) and Singlets (nodes). The biomarkers shown were chosen because of their high activity across patients. Modulator Singlets are octagon nodes (cytokines, and n.a.) and Source Singlets are circle nodes (cells). The magnitude of edges and nodes are sized according Doublet and Singlet total Occurrence Count values. The Singlet value for each is included at its node. The same scale is used for all flow diagrams. The highest valued cellular Source (10) and cytokine Modulator (15) biomarkers as well as one node, “n.a.” representing the linear, non-modulated Quad relationships are included in each flow diagram. “All counts” include all 133,000 Quad relationships for biomarkers in the dataset summed. Green edges and positive values are the cancer values that are greater (more active) than healthy. Red edges and negative numbers represent Doublets and Singlets that are higher in the Healthy group. **a** Average Healthy Cohort with all Quad Occurrence Counts, **b** Average Cancer Cohort with all Quad Occurrence Counts, **c** Healthy greater than Cancer, The difference between the Quads that are greater in Healthy as compared to Cancer, **d** Cancer greater than Healthy, The difference between the Quads that are greater in Cancer as compared to Healthy, **e** Cancer minus Healthy, the overall Quad difference by subtracting the data of healthy volunteers from cancer patients across all counts

CICD begins to reveal the subtle differences that are masked by the commonalities in all donors (cancer and healthy). A main goal of CICD is to uncover the specific networks of active interactions in cancer patients that contribute to their clinical status, relative to healthy donors. The presented flow diagrams visualize the Modulator/Source Doublets and their total Occurrence Counts for both the Doublet and Singlet summations (Fig. 6). Each flow diagram begins with a Quad dataset that is reduced to Doublets (edges) and Singlets (nodes). As explained previously with Table 1, the patterns of Occurrence Counts repeat for all Targets and LSVs across a specific Doublet. Therefore, it can be inferred that active Doublets affect all biomarker Targets to varying degrees, the larger the total Doublet Occurrence Count value the greater the effects on all its biomarkers Targets. The Targets and LSVs of each Doublet provides additional insight and is an area of continued analysis however, is beyond the scope of this paper.

**Table 2:** Comparison of current math models versus CICD model

	Current modeling approaches	CICD modeling
Modeling equations	Ordinary differential equations	Ordinary differential equation
	Delayed differential equations	
	Partial differential equations	
	Stochastic differential equations	
	Agent based models	
	Cellular automata	
	Multiscale/hybrid	
Equations	Multiple combinations	One large matrix equation
Parameters	Multiple, limited by availability	Unlimited
	Measured experimentally and/or estimated theoretically	All are computed
		All possible time varying parameters are contained within the unknown value in the Kolmogorov–Gabor Polynomial
Biomarkers	Limited, most less than 10	Expandable, currently 50
Data Measurement	Measured experimentally and/or estimated theoretically	Sequential daily blood draw
Number of Biomarker Relationships	Limited by current knowledge	Currently 28,605 including all biologically possible relationships
Biomarker Relationships Used	Only known relationships are modeled	All biologically possible relationships can be included
Results	Deterministic or Probabilistic	Deterministic
	Generalized and/or Individualize relative to parameter and patient data availability	Individualize to patient data measurements

A side-by-side comparison highlights the advantages of the flexible CICD approach. These advantages include one simple expandable equation, no estimated parametrization, large numbers of biomarkers and multi-dimensional relationships can be included, and an individualized model of their own immune system generated directly from their own data. As compared to the generalized, parameter dependent modeling found in the current literature

To provide an initial comprehensive view of the CICD resultant data, two cohorts, Healthy or Cancer were averaged across each unique active Quad (133,000 total) generating an average Cancer and an average Healthy CICD Quad dataset. These two representative datasets are compared to highlight the information obtained via CICD modeling (Fig. 6). Both complete Quad datasets Fig. 6a, b and three Quad comparison datasets Fig. 6c, d, e are portrayed (note, dominant signals of MCP-1 [CCL2], IP-10 [CXCL10], IL-8 and CD11c +). For each cancer patient and healthy volunteer, the same complete flow diagrams are generated and found in Additional file 2.

**Fig. 7:**
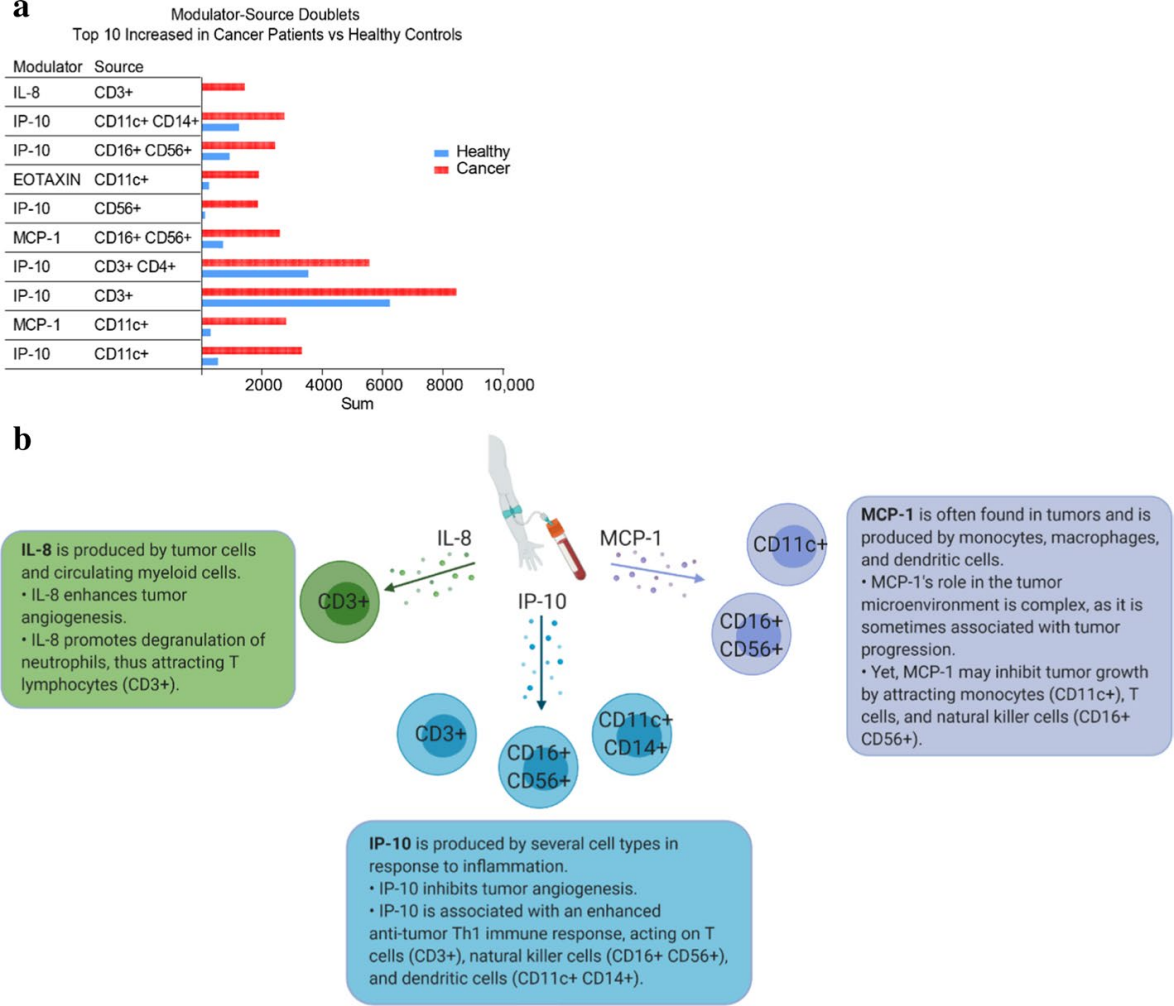
Top modulator-source doublets enhanced in cancer patients compared to healthy controls. **a** From CICD analysis, the ten modulator-source doublets with the greatest difference between cancer patients and healthy controls are shown. **b** The top modulator chemokines in cancer patient peripheral blood are IL-8, IP-10, and MCP-1, which play a role in cancer immunity

The prominent Doublet activity levels (edges) are emphasized in Fig. 7. The biggest differences found between cancer patients and healthy volunteers were modulated by MCP-1, IP-10, and IL-8, seen amongst the top 10 modulator-source doublets enhanced in cancer depicted in Fig. 7a. These chemokines were found to modulate several types of immune cells, including T cells, natural killer (NK) cells, monocytes, and dendritic cells (DC) (Fig. 7b).

The CICD model’s Quad dataset and resultant flow diagrams portray many of the biology’s accepted fundamental truths. The immune regulatory system is highly redundant and tends toward a relatively stable equilibrium among these multiple interdependent elements as seen through the multiple CICD defined relationships driving specific biomarkers activity. This homeostasis is maintained by numerous underlying physiological processes, many that can be possibly defined through CICD. The homogenous and heterogeneous characteristics of the system is modeled through the multiple identical relationships, yet different values found in the CICD datasets between both cancer and healthy cohorts as well as among individuals. This is first observed when examining the full Quad and Triplet data, the same relationship yet difference values are found across patients and cohorts that is then visualized by the Modulator-Source Doublets flow diagrams. The calculations of the subtle differences underneath the seemly similar raw data found with CICD provides the individualized modeling needed to unlock clinical pathways to tailor treatment to a patient’s specific immune status.

## Discussion

The main premise of this work is that singular biomarker discriminators (single measurement at a single time) have not been successful in describing the interconnected nature of different regulators of immune function in peripheral blood of humans with cancer. One of the main challenges in clinical cancer immunology is that, despite multiple efforts, it has been difficult to define a single measurement/predictor of immune cell function that encompasses the state of immunity in a patient with cancer. This has been particularly challenging in efforts of developing predictive biomarkers for cancer immunotherapy, where the only biomarker of outcomes for anti-PD1 therapy (tumor associated PDL1 expression) continues to be highly debated and remains of notable but limited clinical value. Part of the reason for this challenge is the acknowledged complexity of the numerous interacting components of human immune system regulation, not all of which play the exact same role in different patients. Thus, we undertook an effort to collectively examine the multitude of commonly used biomarkers of the state of systemic immunity, analyze their interactive properties based on their temporal variability and mathematically define (CICD) the overall state of systemic immunity, shared by cohorts of individuals, but, attained via similar but not identical pathways. Herein we present an integrated analysis of a multitude of pre-selected “biomarkers” of immunity that describe the difference between the state of systemic immunity in cancer patients, versus that of healthy volunteers.

The data reveals a dominant signal by two chemokines MCP-1 (CCL2) and IP-10 (CXCL10) that are known to affect multiple cell types, as suggested by our data, with a somewhat more prominent impact on CD11c + cells in peripheral blood. These data are consistent with our appreciation of the role of chemokines in advanced cancer [27] as well as the putative role of CD11c + immune cells in maintenance of chronic inflammation [28]. When looking at the biggest differences between cancer patients versus healthy controls, we noticed that MCP-1, IP-10, and IL-8 were amongst the top modulators in cancer, acting on several types of immune cells, shown in Fig. 7. These results suggest that the main mechanisms of immunologic homeostasis of cancer are driven by a subset of chemokines for which there is published data demonstrating their role in cancer immunity. MCP-1 (CCL2) and IL-8 (CXCL8) are chemokines known to allow tumor progression by promoting tumor angiogenesis [29, 30]. However, MCP-1 is also known to attract monocytes, NK cells, and T cells [31, 32]. Furthermore, IL-8 may also modulate the immune system in a favorable manner, as it has been shown to act as a chemoattractant for T cells [33]. In contrast to MCP-1 and IL-8, IP-10 (CXCL10) is able to inhibit angiogenesis [34, 35]. IP-10 is secreted by cells in the context if inflammation and promotes a Th1 immune response involving T cells, NK cells, and DCs [36–39]. Thus, the modulator-source interactions that CICD associated from peripheral blood samples aligns with observations in the literature.

Hence, in a purely mathematical way, the current analyses have identified a plausible network of cells/cytokines (Modulator/Source) that play a role in sustaining the state of immunity in advanced cancer. This provides a starting point to a more focused analyses regarding the role of these dominant immune mediators in patients with cancer, and offers an analytical tool that may be used to test additional biomarkers, relevant to MCP-1 (CCL2) and IP-10 (CXCL10) and in more detail describe the network of interactions in the absence or presence of therapeutic interventions, and resultant clinical outcomes, possibly identifying novel therapeutic targets and/or prognostic/predictive biomarkers.

At the current juncture, the CICD model is only asking what biomarkers (measured in peripheral blood only) are working together regardless of previous knowledge of their function. CICD observes the interaction behaviors of the immune system, without considering the many possible underlying mechanisms that combine to produce an active interaction. Identification of the sub-groups (sub systems) of interacting biomarkers that discriminate cancer patients from healthy volunteers, elucidate critical differences in the states of immune homeostasis that may be relevant to the natural history of the malignancy as some of these elements of homeostasis (PD1 + immune cells) are targets of modern cancer immunotherapy.

A fundamental feature of CICD is the Knowledge Model. Unlike common approaches that focus on a limited number of biomarker interactions, CICD’s goal was to include all possible immune biomarker interactions to begin to unravel the immune regulatory complexity. The plasticity of the Knowledge Model allows future studies to expand and/or modify its interactions and biomarkers to assist in revealing biological interactions thru mathematics. The Knowledge Model is the unique feature of CICD that allows a flexible analysis of biomarkers’ interaction network without any experimental constraints based on previously investigated interactions. The innovative Knowledge Model lists all biologically possible causes or relationships between the biomarkers studied, whether or not they have previously been studied. Future studies look to modify the Knowledge Model by either narrowing or expanding the number and/or type of relationships to further explore the immune interaction network are currently under way. The flexibility of the Knowledge Model is fundamental in uncovering the complexity of the system.

The second essential requirement for CICD is the sequential collection of peripheral blood samples. This provides the necessary information to specifically assess an individual without any theoretical estimation of parameters. As seen reflected in the CICD results data, the raw data also contains similarities and differences among participants. Through CICD, the specific underlying causes that manifest in these observed biomarker oscillations can be quantified. Future studies will focus on determining the number blood draw data points required for optimal analysis reliability.

It is well established that biological systems are complex and redundant systems that will effectively maintain functionality if a failure occurs within the system. The human immune system illustrates this concept through numerous immunological mediators (biomarkers) working side by side to protect the body from disease. This presents a most interesting math modeling challenge to engineers that expect laws of behavior to be known exactly and solutions to be predictable, unique and verifiable. Rather than hypothesize ODEs to solve, a Knowledge Model is used to specify equations for all possible biomarker interaction response, and a set of characterization parameters defined as a vector of unknowns. The net result is an underdetermined system with far fewer equations than unknowns [40]. Such systems have an infinite number of equally valid solutions, solvable via SVD methods and the pseudo-inverse. It is well-known that for this class of SVD problems, singular values are unique while singular vectors are not. Recent work [41–43] is enhancing SVD capability to compute one solution that is least effort and which satisfies additional modeling objectives and application needs. Through the SVD algorithm, one of an infinite number of equally valid and least effort solutions is computed, inherently providing a model of the redundant, regulatory/system homeostasis mechanisms of the human immune system. The redundancy and robustness utilized by the immune system to maintain functionality despite changes in the system due to disease can now be mathematically modeled via the power of SVD.

A comprehensive literature review of current biological math modeling techniques was completed to assess CICD’s approach [2–7, 13–18, 44–57]. A side-by-side comparison of several aspects of current techniques vs. the alternative CICD methodology is presented in Table 2 to elaborate the advantages of CICD. Most current models tend to speculate on a limited subset of potential interactions and attempt to recover model parameters to match a set of observed data, a predicative forward modeling strategy. CICD exploits the uncommon reverse modeling strategy to determine underlying causalities that is expressed in the clinical data to understand the complexity of the immune system. Overall CICD does not assume a postulated mathematical model; instead it measures outcomes in the sample data which provide critical insights into the generally unknown internal working of the immune system response. Mathematical modeling converts assumptions into conclusions with certainty always relative to choice of assumptions [44]. To have coincidence in conclusions it has been necessary for current models to keep to simple and limited number of assumptions. These low-resolution models provide limited descriptions of the immune system and the need to shift to dynamic comprehensive modeling is apparent [45].

Current math models utilized multiple mathematical approaches such as various differential equations and agent-based modeling to model a limited number of specific biological assumptions by measurable or estimated parameters. CICD revises this current structure by allowing thousands of assumptions, both known and unknown relationships to be quantified using one generalized ODE equation with only clinical data and without parameterization. Current models’ results are highly reliant on multiple parameter values. A major advantage of CICD is that its algorithms and thereby its results are not dependent on the accuracy of estimated or theorized parameters. CICD allows all underlying parameters to be represented by the unknown value. By isolating the values for all parameters, the computational burden of the model is dramatically reduced, and the system of equations simplified, thereby enabling CICD analysis to focus on the information obtained via SVD. This vector of calculated parameter values is another avenue for investigation in future studies. An additional unique and superior characteristic of CICD modeling is the ability to change how many and which specific biomarkers will be analyzed without an assumed knowledge of a biomarker’s mechanism in the immune system. The flexibility of CICD is boundless and enables the ability to tailor the biomarkers and relationships to the focus of the study. CICD results are based on a patient’s blood draw measurements alone. This individualized immune math model can potentially unlock tailored treatment plans for a person’s specific clinical status and the ability to monitor their immune system over the course of treatment as well as beyond. CICD’s application of the SVD algorithm in this unique way is the foundation of its approach. The SVD algorithm has enabled considerable advances in the modern world, such as facial recognition [58]. Now CICD uses the power of SVD to continue to advance understanding of the biological world.

## Conclusions

CICD equations expose underlying causes, previously imperceptible nonlinear coupling biomarker interactions that together affect the observed populations of the biomarkers in the blood, thereby providing an insight rich snapshot of an individual’s state of immune homeostasis. Its main objective is discovery, to help lead to improvements in the efficacy of existing immune therapies (patient selection; drug combinations) and insight into new therapeutic targets that may significantly reduce the time to discover new therapeutics capable of meaningful clinical impacts.

This new perspective of biological modeling from the current predictive approach to the CICD descriptive approach unleashes vast potentials not only in understanding of the complexity of the immune system but continues the pathway to individual treatment plans. Possible applications of CICD are many, not only in other cancers but in other diseases as well, utilizing this model of complex, redundant, ill-defined, biological systems. This collaboration between biology and engineering disciplines exemplifies how “the purpose of computation is insight, not numbers” is pivotal in the quest for a cure [59].

## Supplementary Information

**Additional file 1. Flow Cytometry and Cytokine Measurement**: Detailed methods used to obtain biomarker measurements.

**Additional file 2. Individual Peripheral Blood Derived Serial Biomarker Data and Flow Diagrams**: A graph of peripheral blood biomarker measurements and flow diagram is provided for each cancer patient and each healthy volunteer. All Quads for the individual are included in each diagram. Graphs are in the same format as Fig. 4. Flow diagrams are in the same format as Fig. 6.

## Data Availability

The data that support the findings of this study are available from Math for Medicine, Inc. but restrictions apply to the availability of these data, which were used under license for the current study, and so are not publicly available. Data are however available from the authors upon reasonable request and with permission of Math for Medicine, Inc.

## Abbreviations

CICD: Cancer immune control dynamics
SVD: Singular value decomposition
ICI: Immune checkpoint inhibitors
ODE: Ordinary differential equation
LSV: Left singular vectors
RSV: Right singular vectors
