# Supplementary material for: Cancer immune control dynamics: a clinical data driven model of systemic immunity in patients with metastatic melanoma
Source: BMC Bioinformatics. 2021 Apr 16;22:197. doi: 10.1186/s12859-021-04025-7 (PMC8052714; doi:10.1186/s12859-021-04025-7)

**Additional File 1**

**Flow Cytometry and Cytokine Measurement:**

Venous blood was collected from melanoma patients and healthy volunteers daily for 10 days in sodium heparin collection tubes. Blood samples were spun to acquire plasma and ficoll gradient was employed to isolate peripheral blood mononuclear cells (PBMC) according to an SOP. Plasma was stored in 1ml aliquots in a -80C freezer for later study. PBMC were washed and immediately prepared for flow cytometry analyses. All the fluorochrome labeled monoclonal antibodies were purchased from BD Biosciences (Franklin Lake, NJ). Appropriate antibody cocktails were mixed and added to cells for labeling. After 30 minutes, labeled cells were washed twice and run by flow cytometry on a Millipore Guava 8HT (Burlington, MA). Flow data was analyzed using Millipore Guavasoft software (Burlington, MA). For lymphocyte populations gates were set on the lymphocyte gate determined by forward and side scatter. From the live lymphocyte gate, monocyte, T-cell, and NK cell subsets were enumerated. For dendritic cells and macrophages, live mononuclear cells were gated from forward and side scatter and Lin+ cells (CD3, CD14, CD16 and CD19) were negatively gated. From the lin- gate, macrophage and dendritic cell subsets were enumerated. All flow data is reported as relative frequency. To determine plasma cytokine concentrations, we used the Millipore 38-plex (Burlington, MA) according to manufacturer’s instructions. Briefly, plasma samples were thawed and 25ul was placed into a 96-well plate with antibody coated magnetic beads and incubated overnight at 4^o^C. Then beads were then washed and biotinylated detection antibodies were added and the plate was incubated at room temperature with shaking for 1 hour. Horse radish peroxidase (HRP) conjugated phycoerythrin (PE) was added to the detection antibody coated beads. Washed beads were then run on the Luminex 200 and protein concentrations were calculated using a standard curve (3.2-10000pg/ml) using Milliplex Analyst software from Millipore (Burlington, MA). Percent CV of replicates and standard curves were used to validate the integrity of each run. Gating strategy of the cell flow cytometry is provided below.

T-cell gating


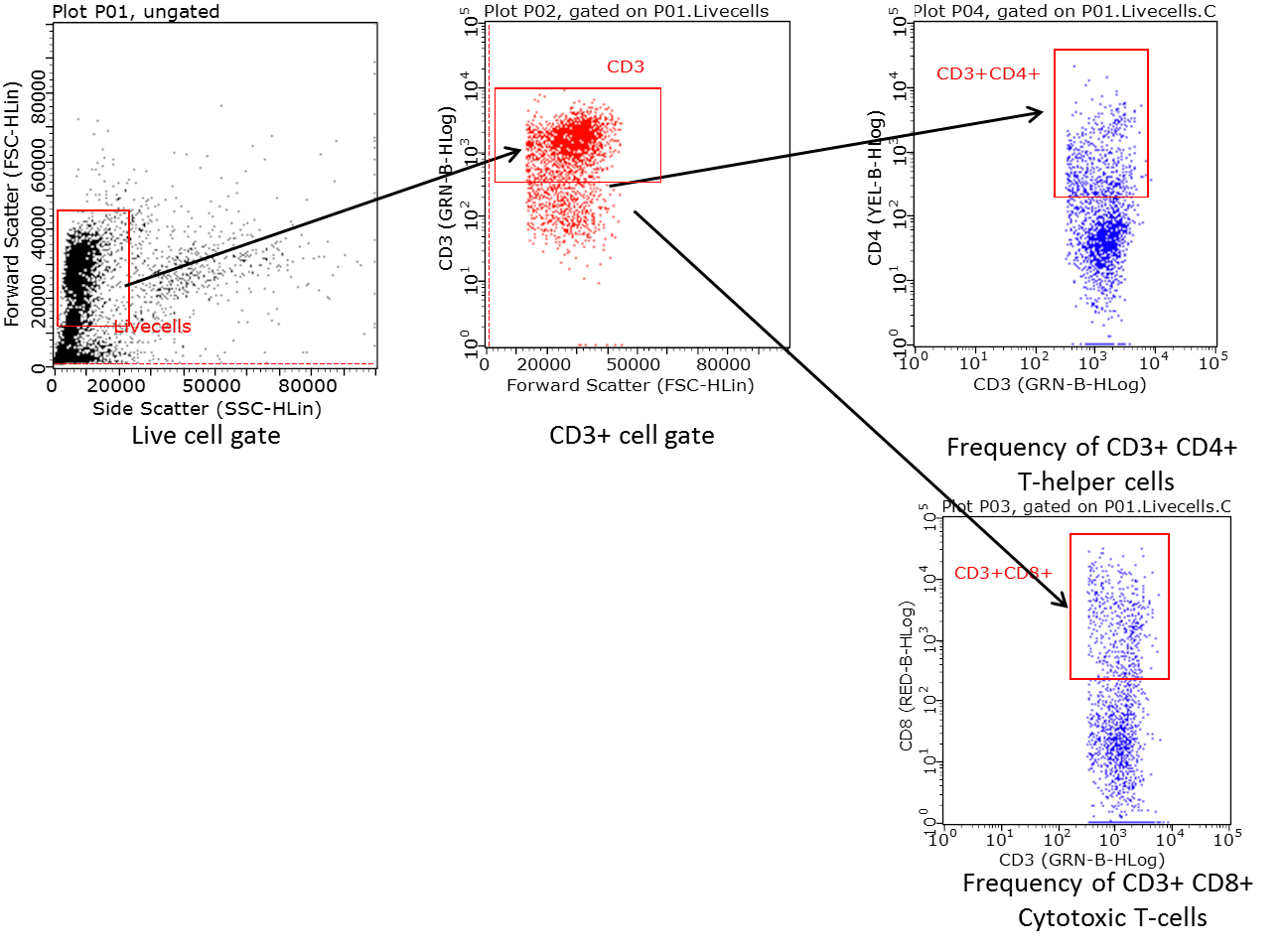


Dendritic Cell Gating


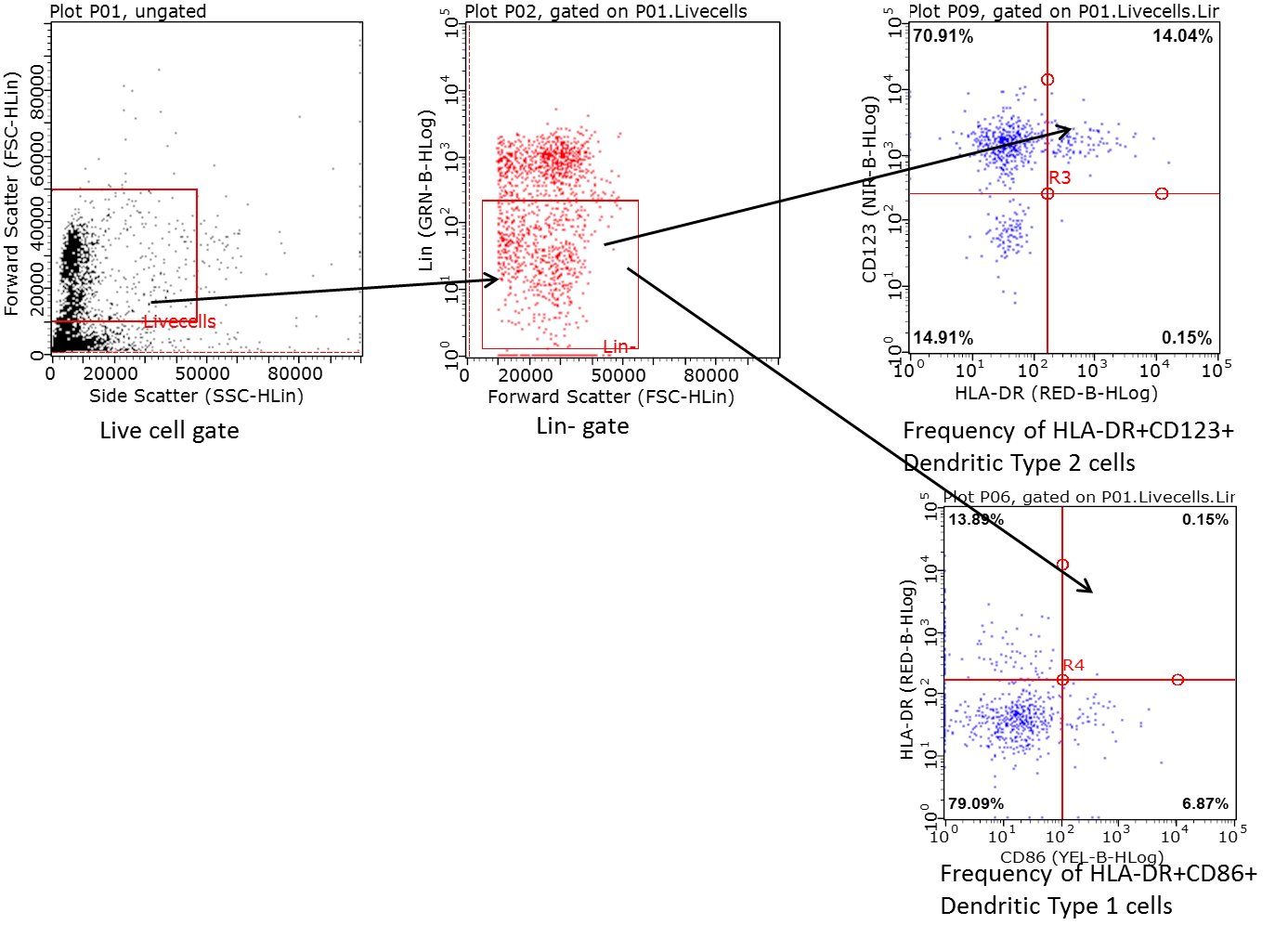

Supplement: Supplementary file 1 — Additional file 1. Flow Cytometry and Cytokine Measurement: Detailed methods used to obtain biomarker measurements. [file 12859_2021_4025_MOESM1_ESM.docx]
