# Supplementary material for: Cancer immune control dynamics: a clinical data driven model of systemic immunity in patients with metastatic melanoma
Source: BMC Bioinformatics. 2021 Apr 16;22:197. doi: 10.1186/s12859-021-04025-7 (PMC8052714; doi:10.1186/s12859-021-04025-7)

Cancer Patient 11: Female, age 77

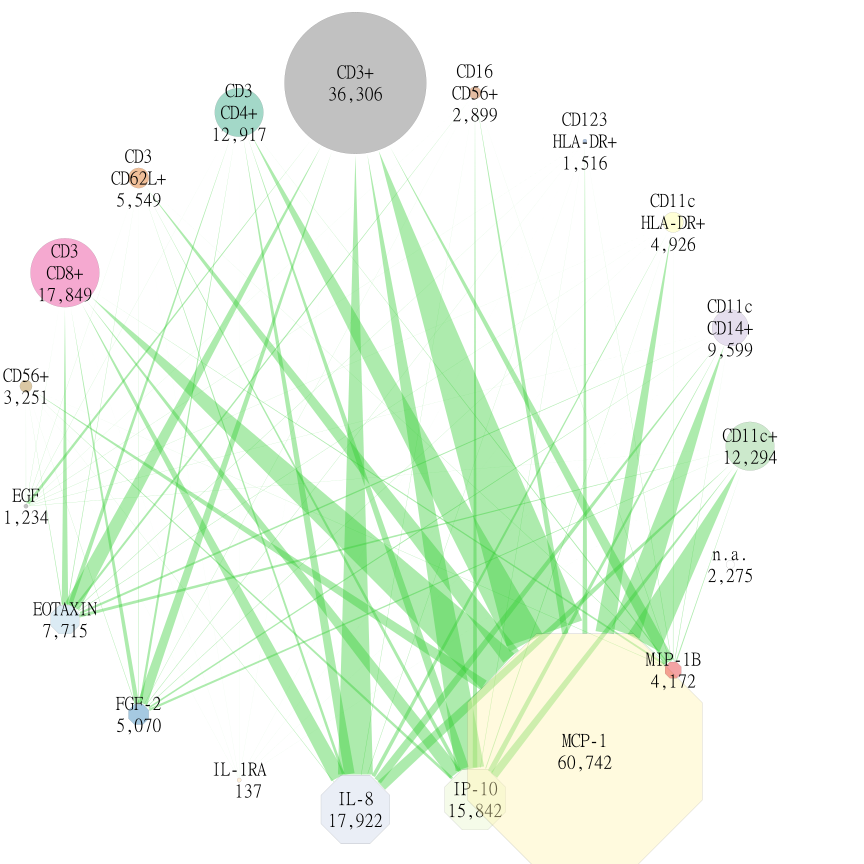

Cancer Patient 12: Female, age 83


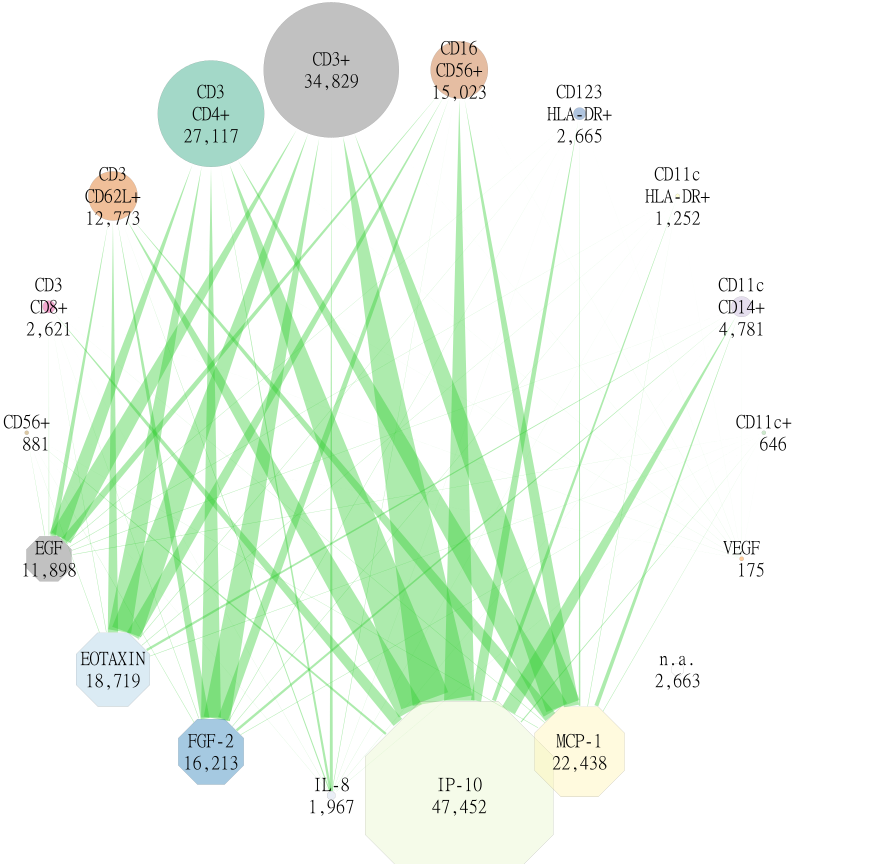

Cancer Patient 13: Female, age 39

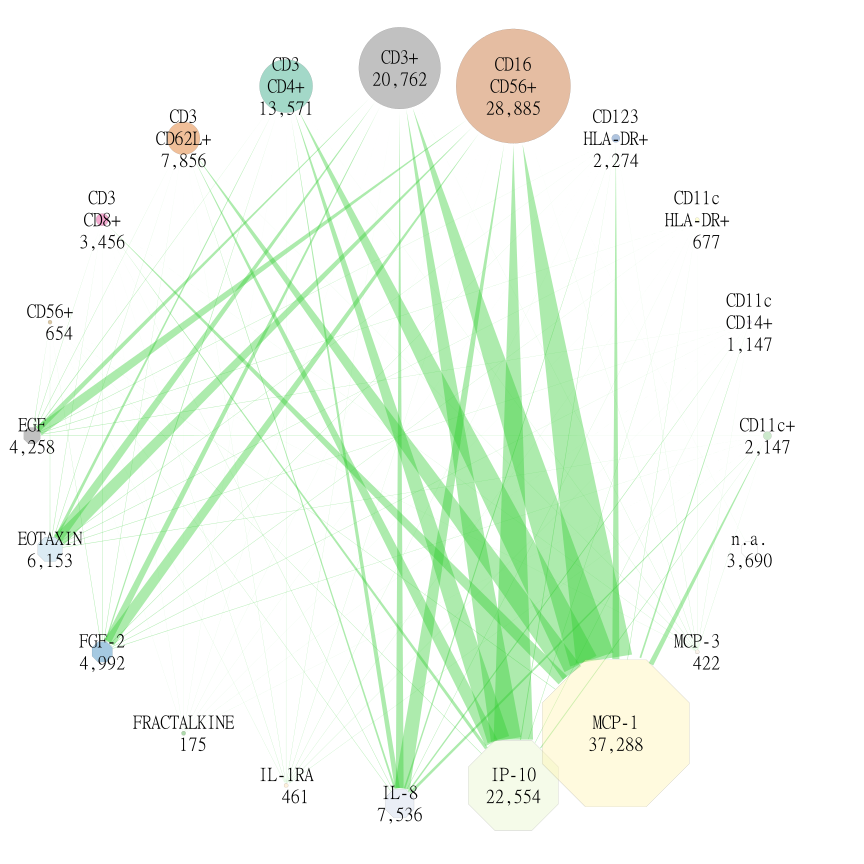

Cancer Patient 14: Male, age 62

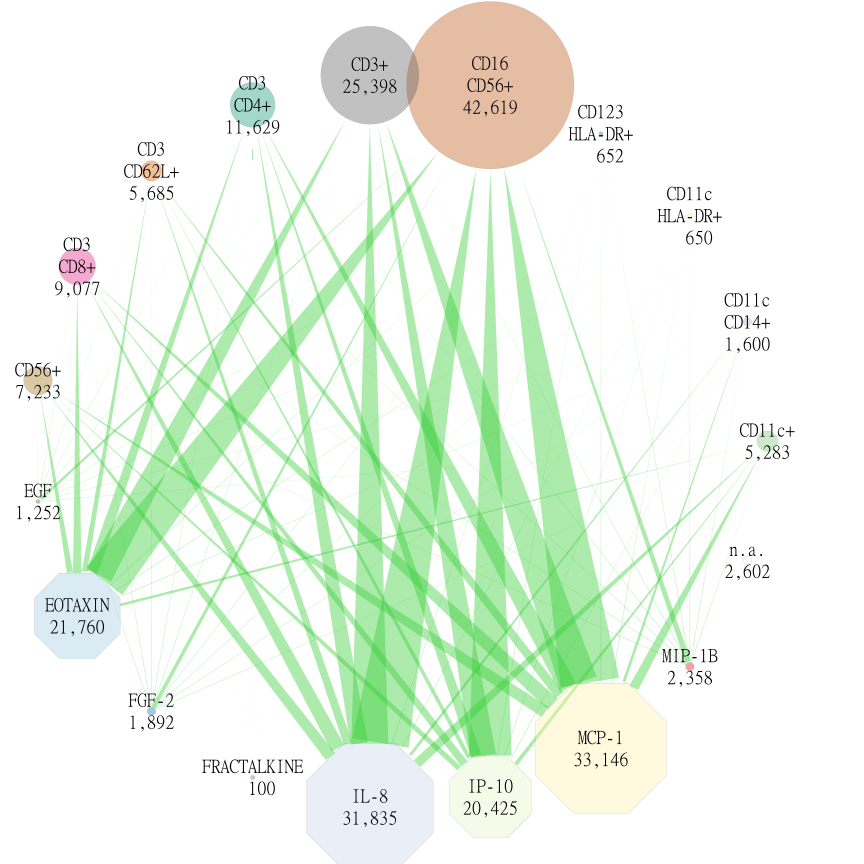

Cancer Patient 15: Male, age 81

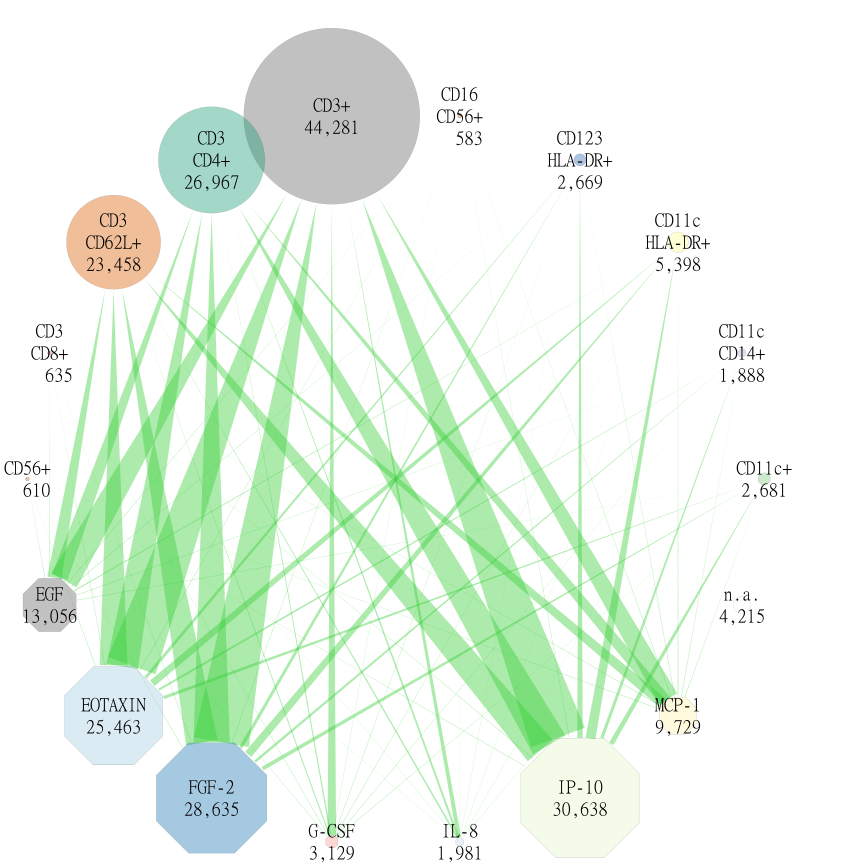

Cancer Patient 16: Female, age 67

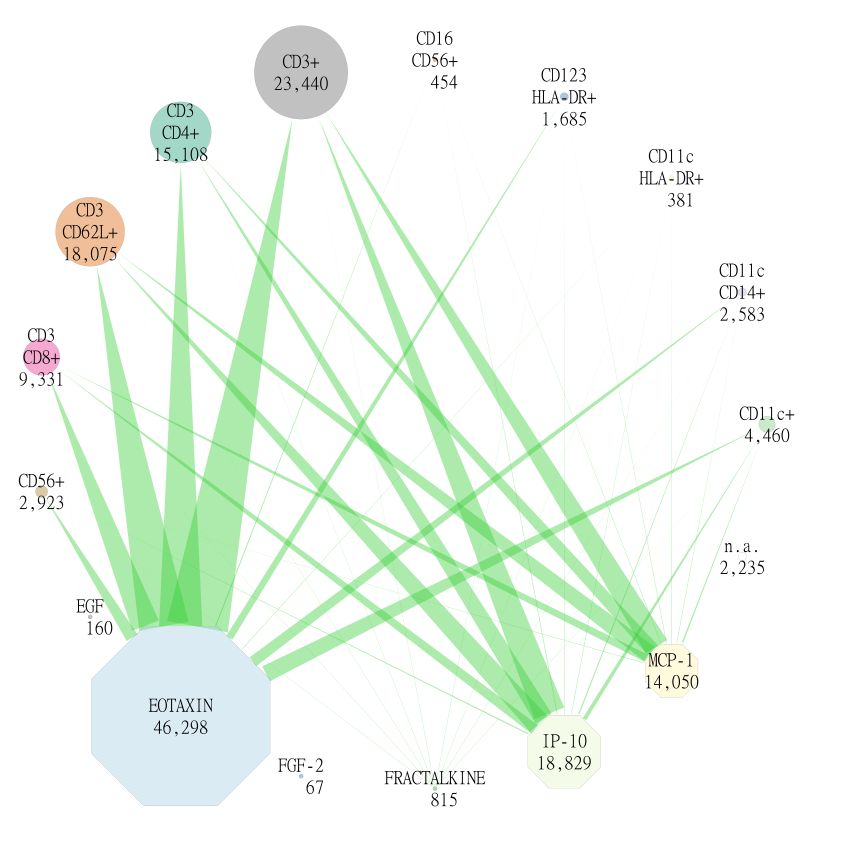

Cancer Patient 17: Male, age 70


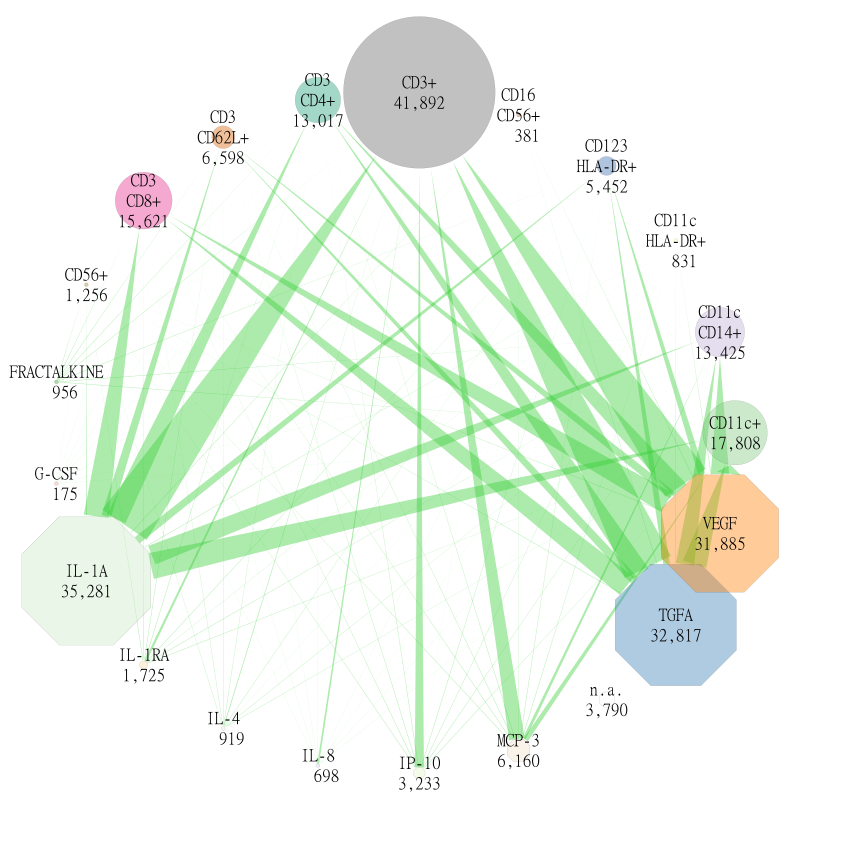

Cancer Patient 18: Female, age 62

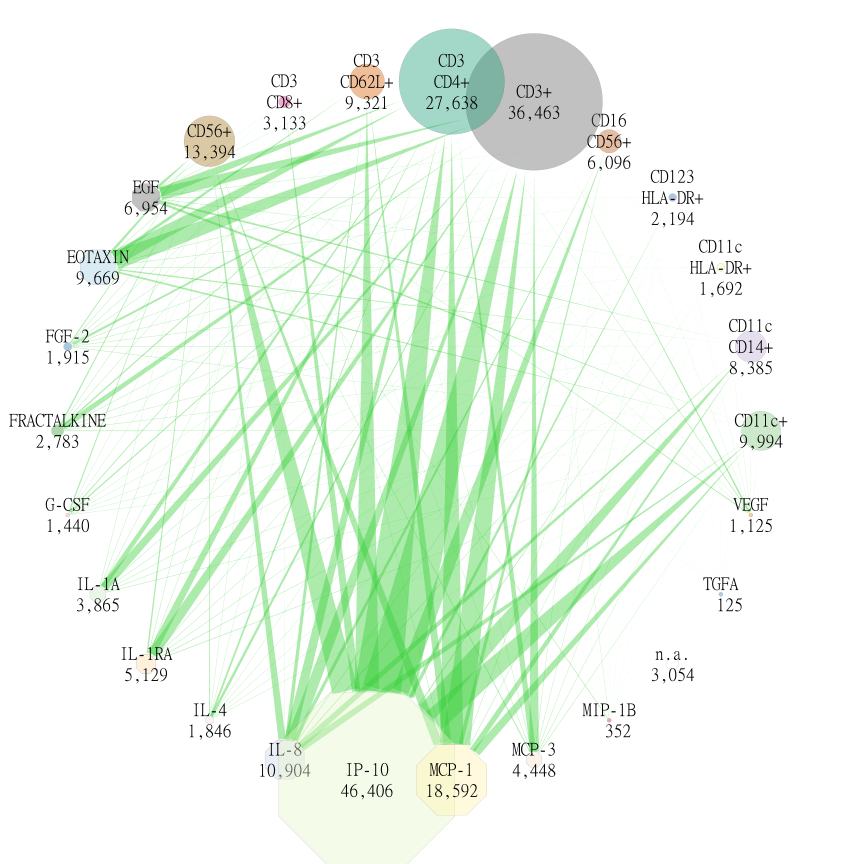

Cancer Patient 19: Female, age 84
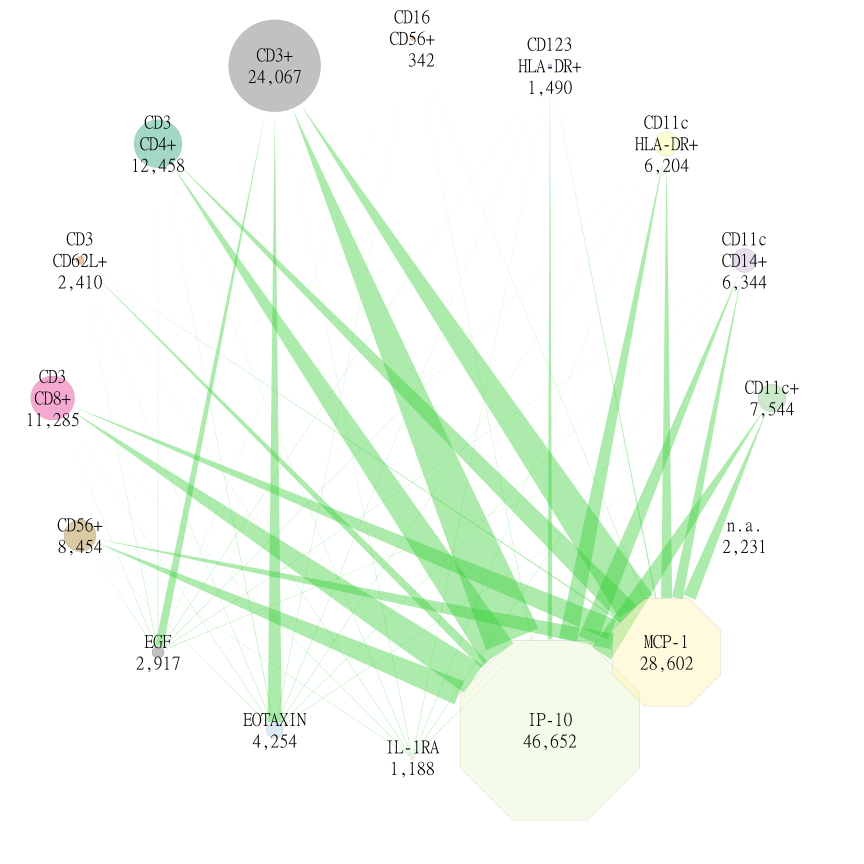

Cancer Patient 20: Female, age 60

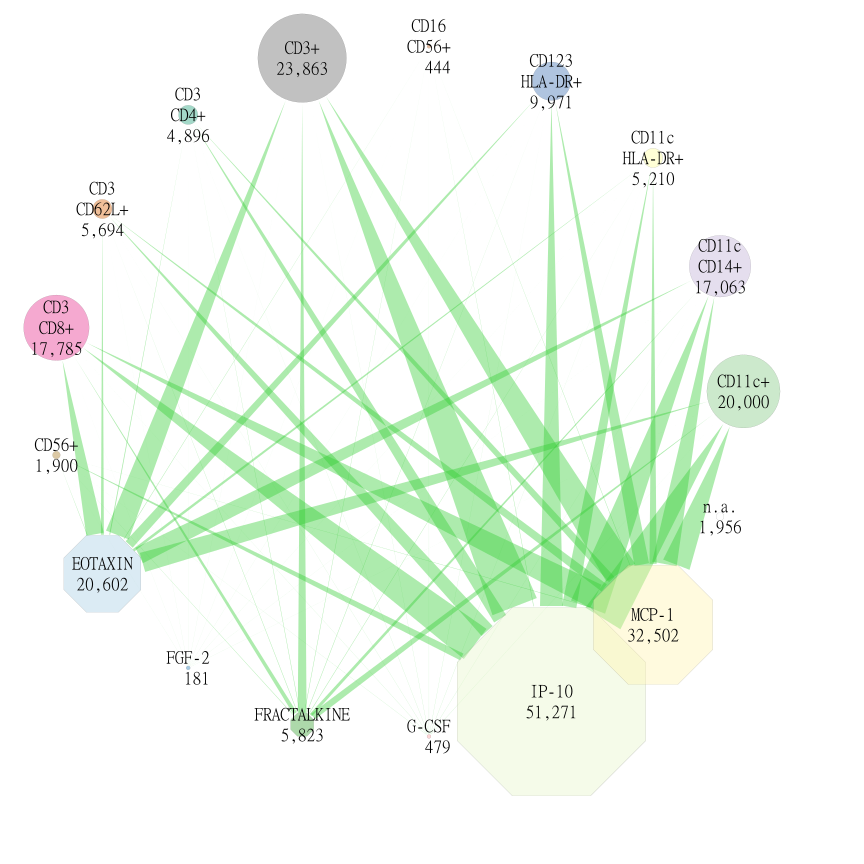

Cancer Patient 21: Female, age 76

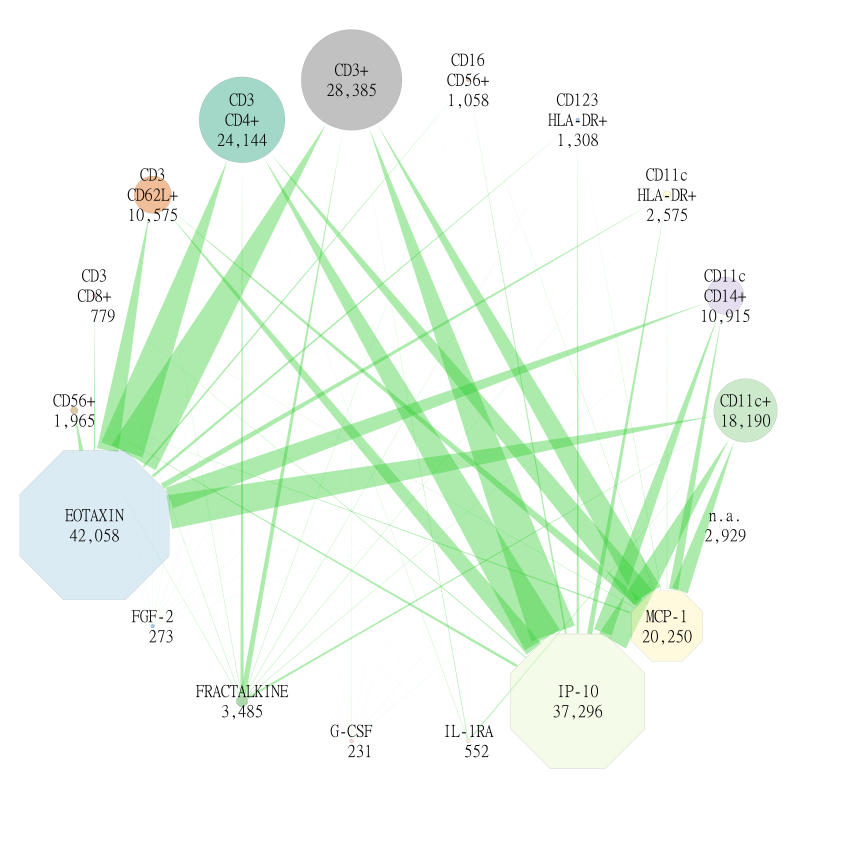

Cancer Patient 22: Male, age 63

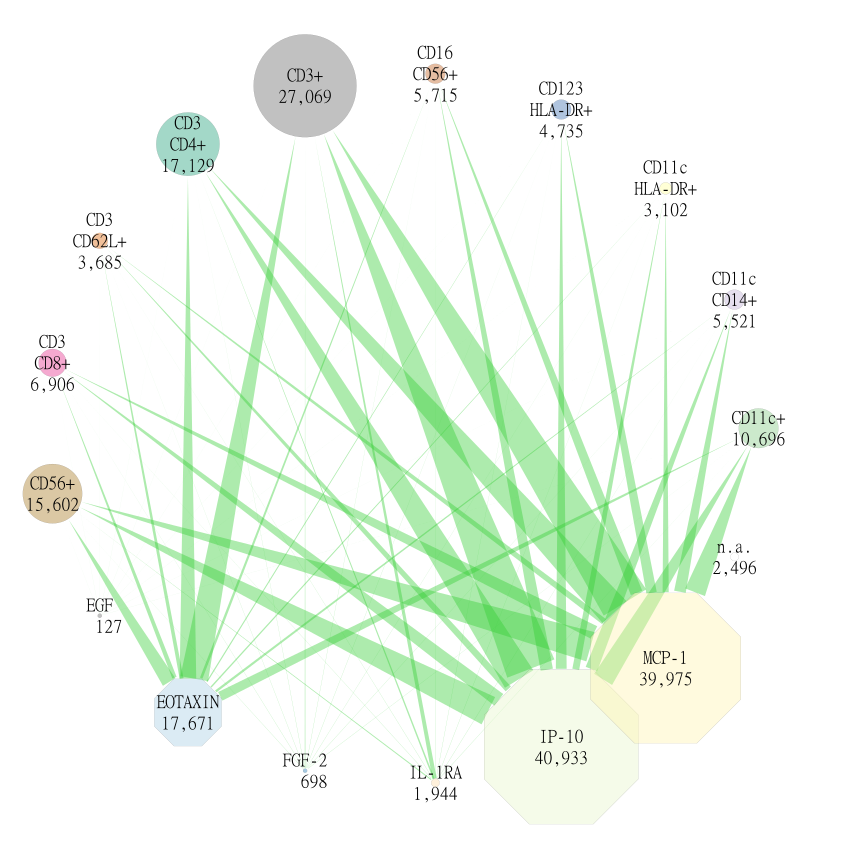

Cancer Patient 23: Male, age 72

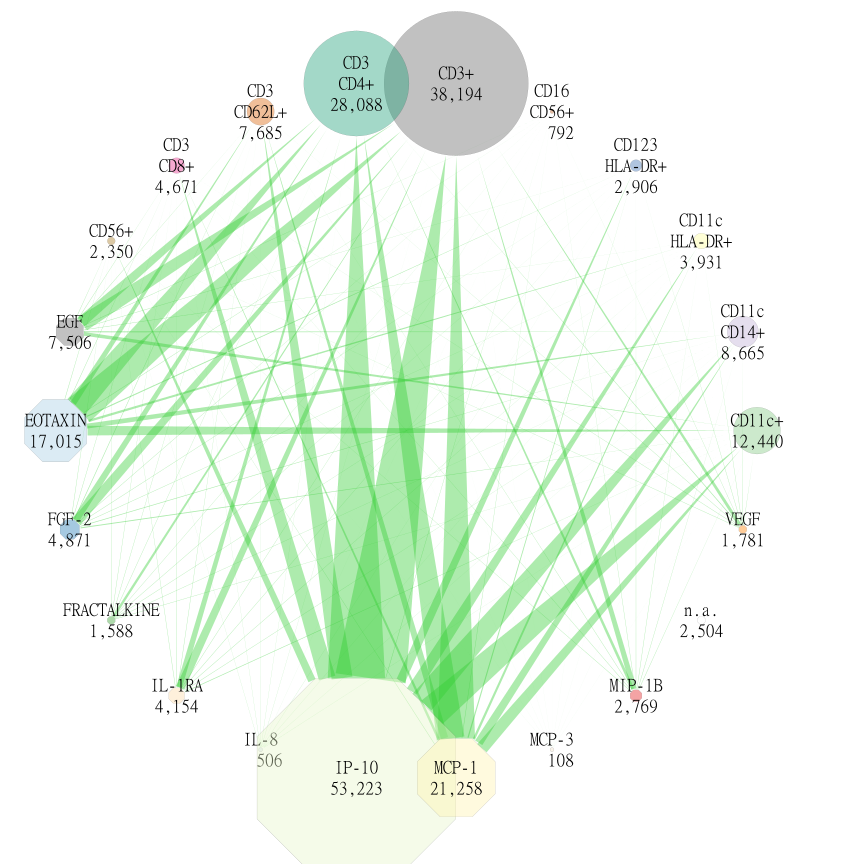

Cancer Patient 24: Male, age 60


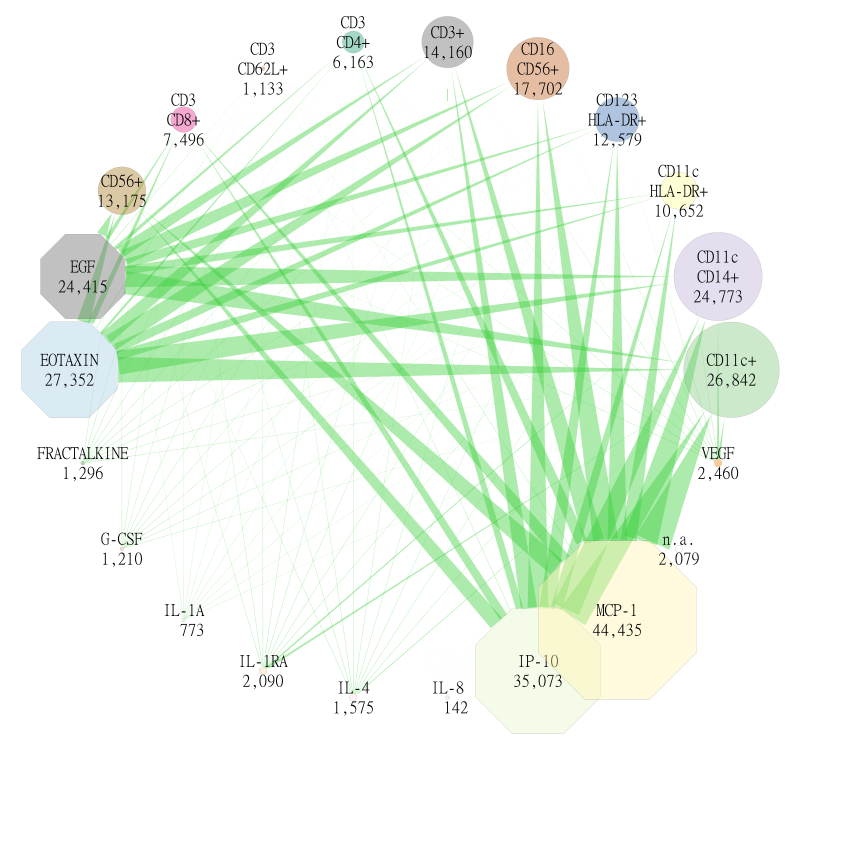

Healthy Patient 1: Male, age 30

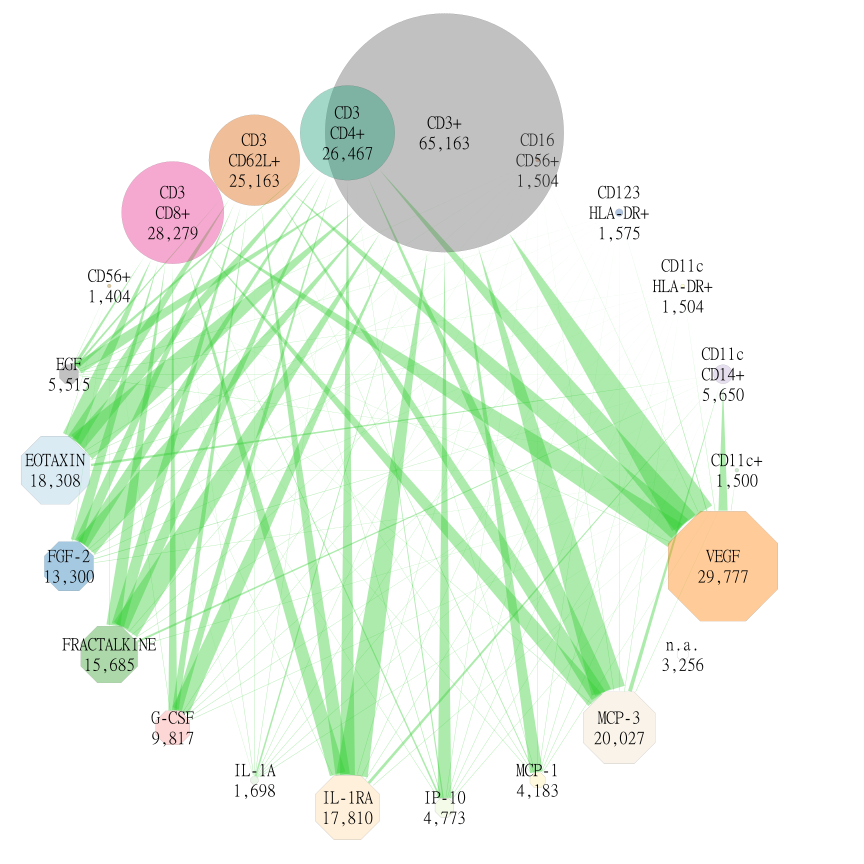

Healthy Patient 2: Female, age 32
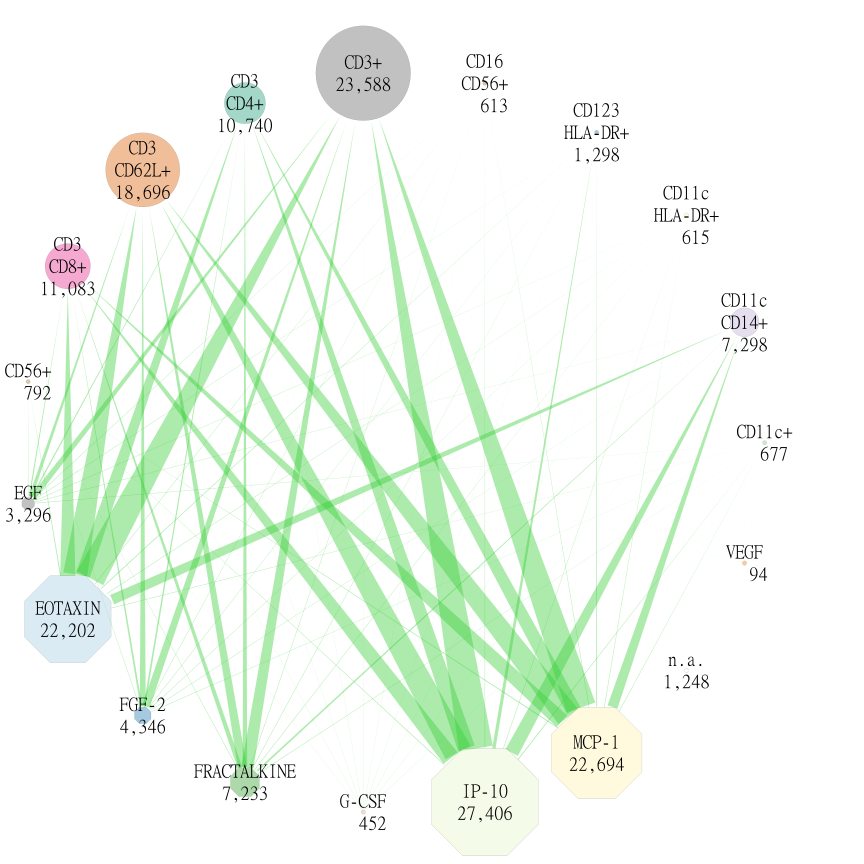

Healthy Patient 3: Female, age 27
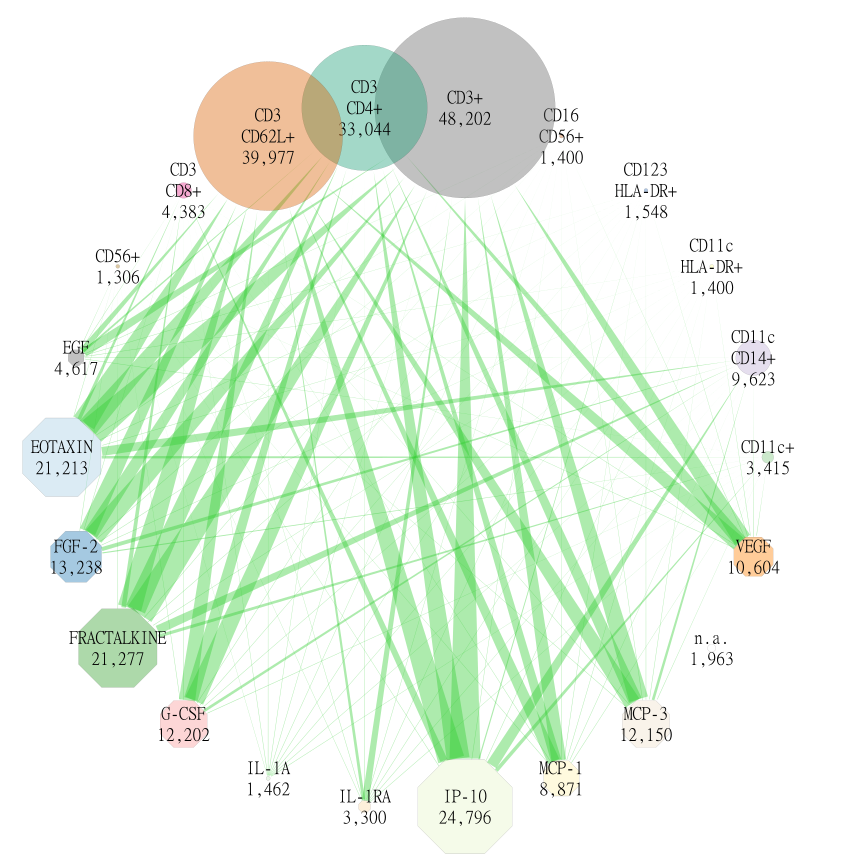

Healthy Patient 4, Male: age 31
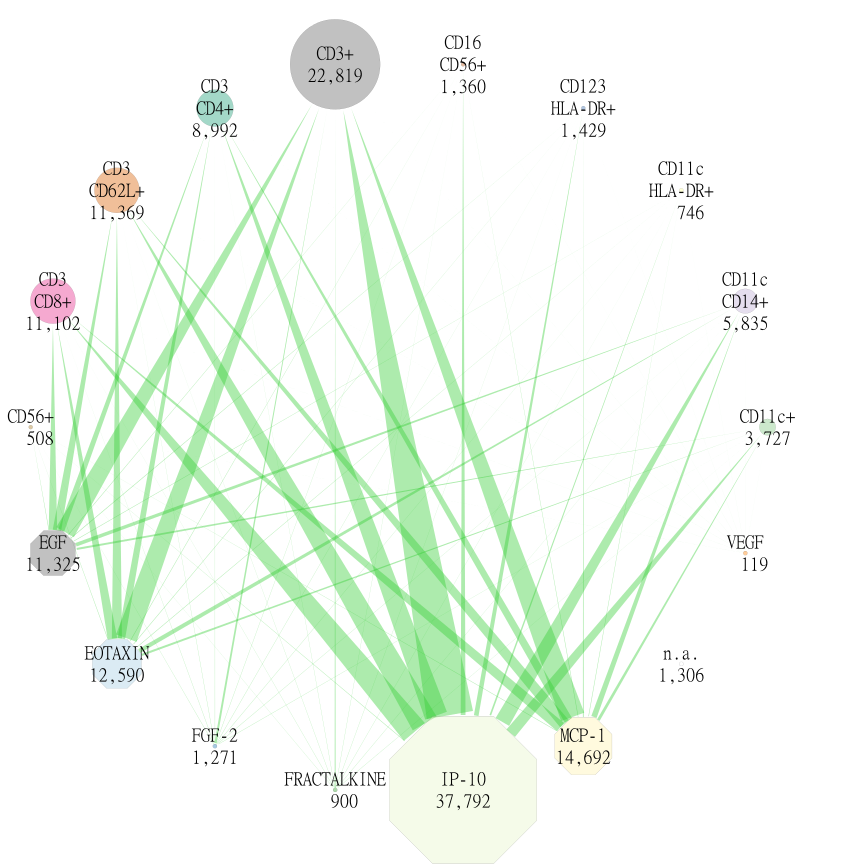

Healthy Patient 5: Male, age 30
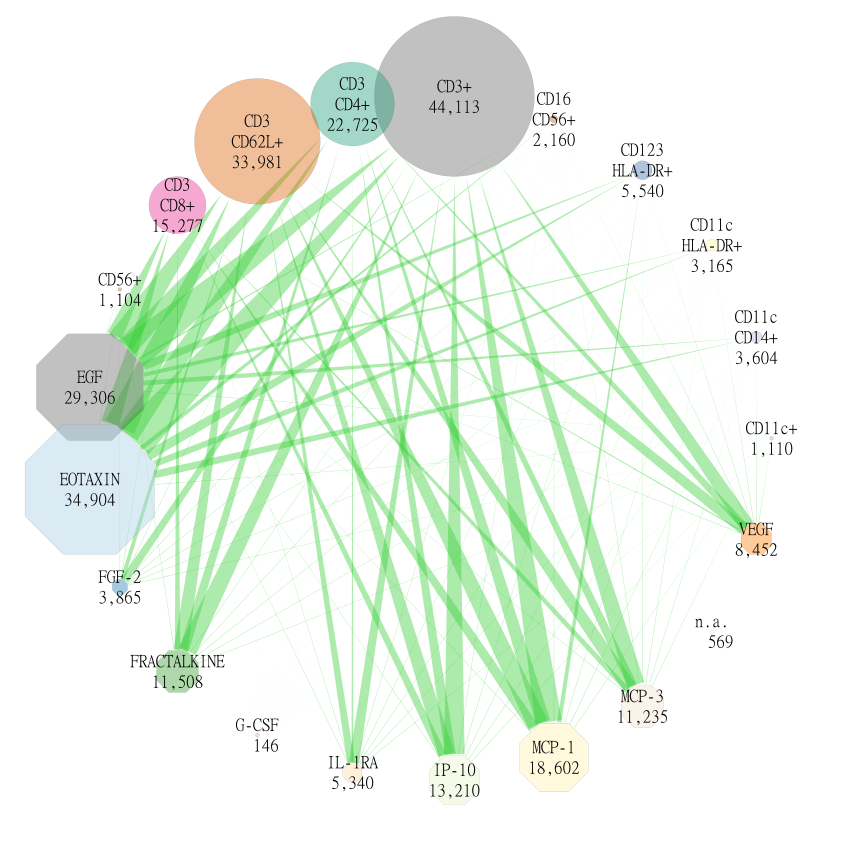

Healthy Patient 6: Female, age 40
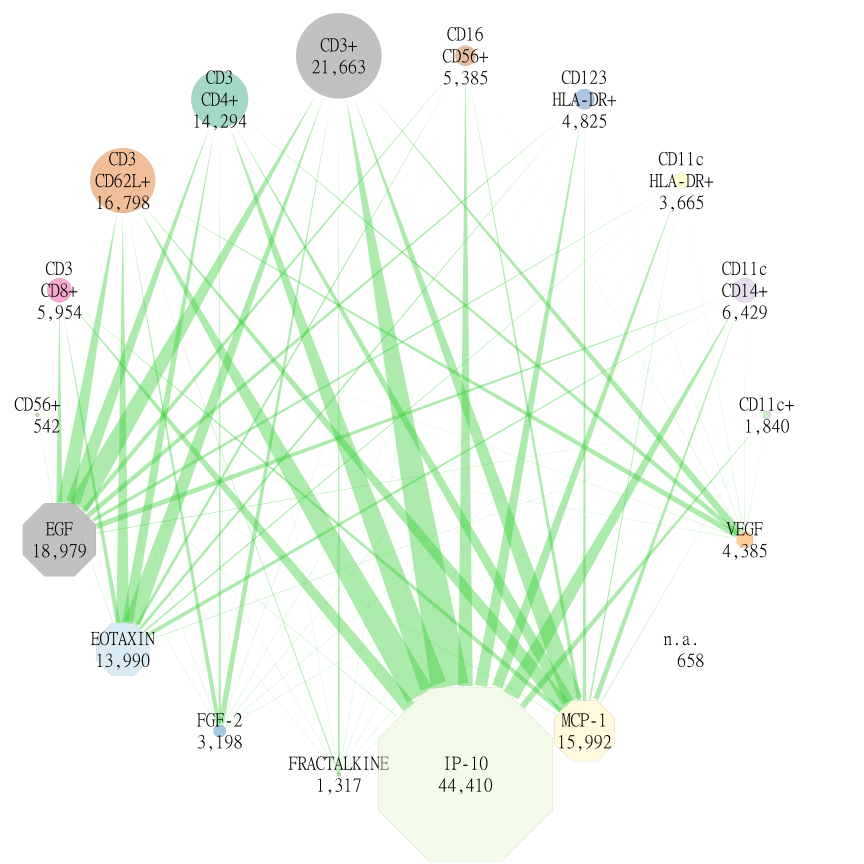

Healthy Patient 8: Female, age 38
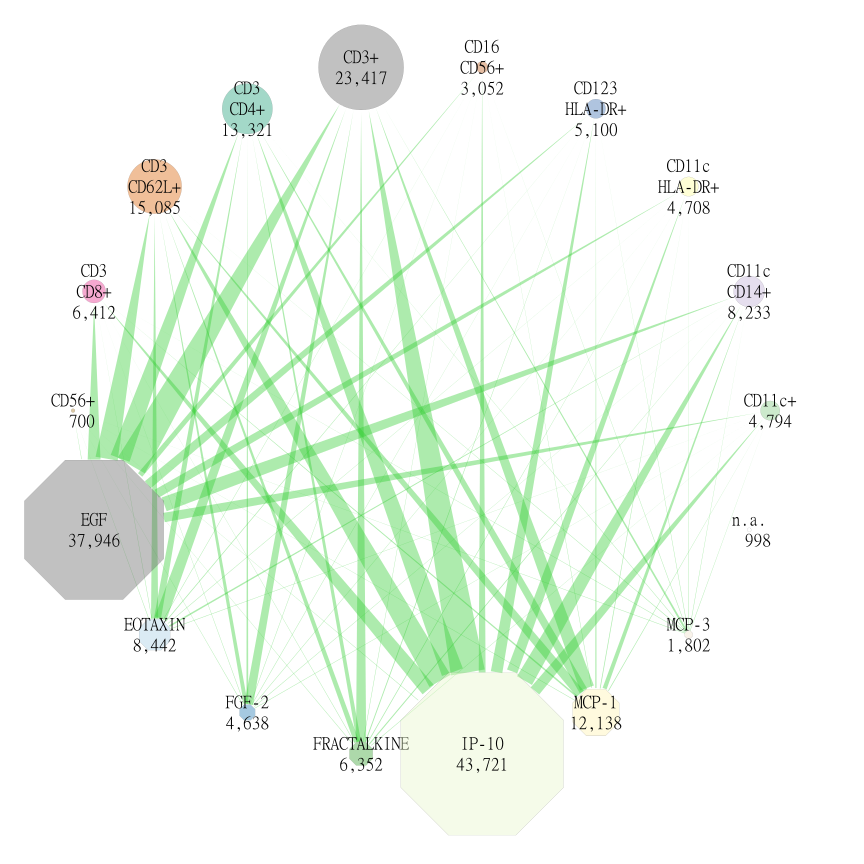

Healthy Patient 9: Female, age 25
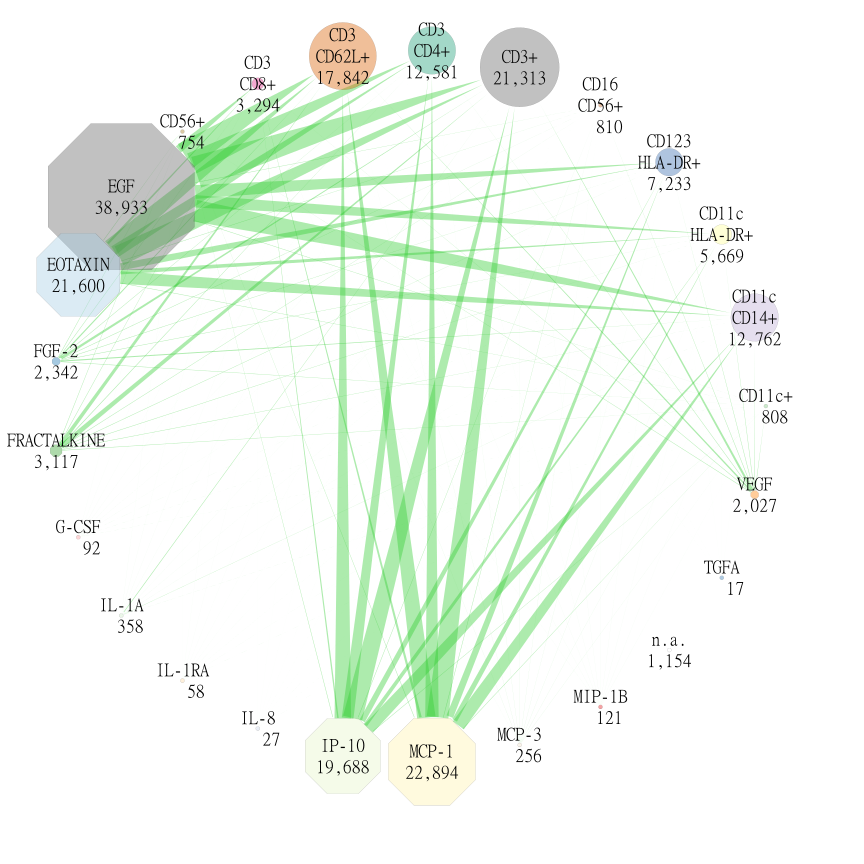

Healthy Patient 10: Male, age 35
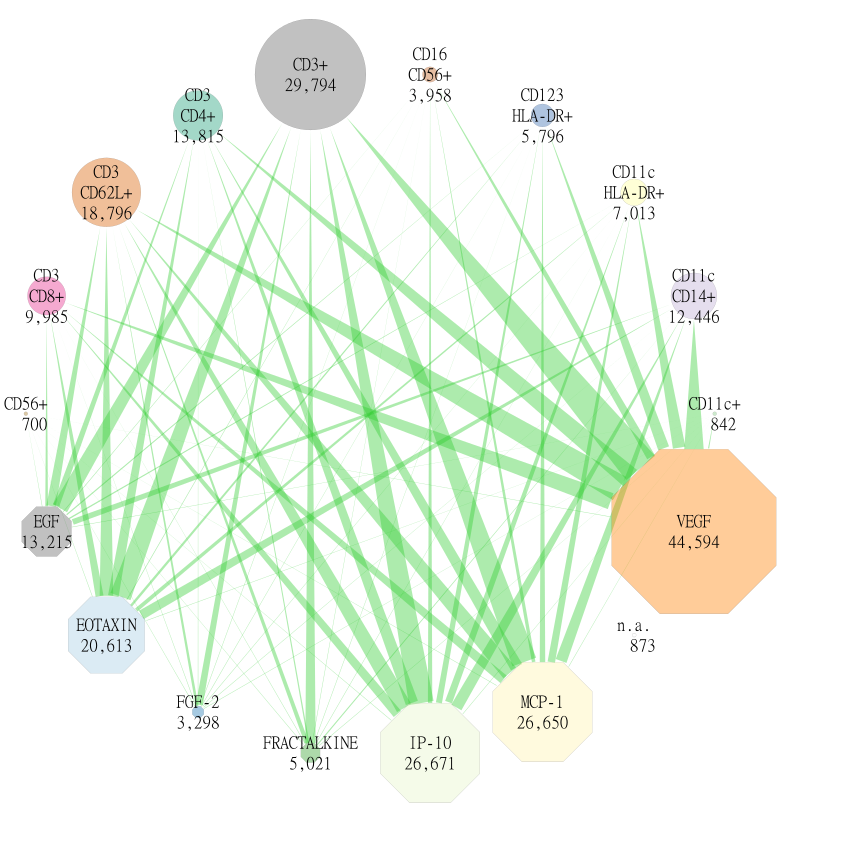

Healthy Patient 12: Female, age 41
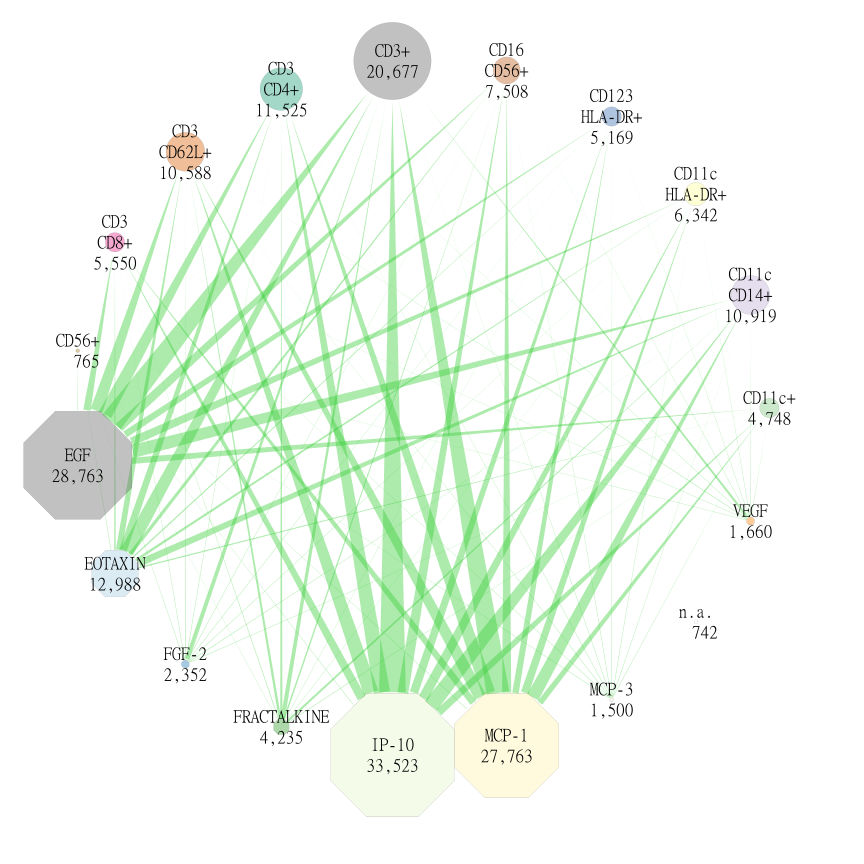

Healthy Patient 14: Male, age 42
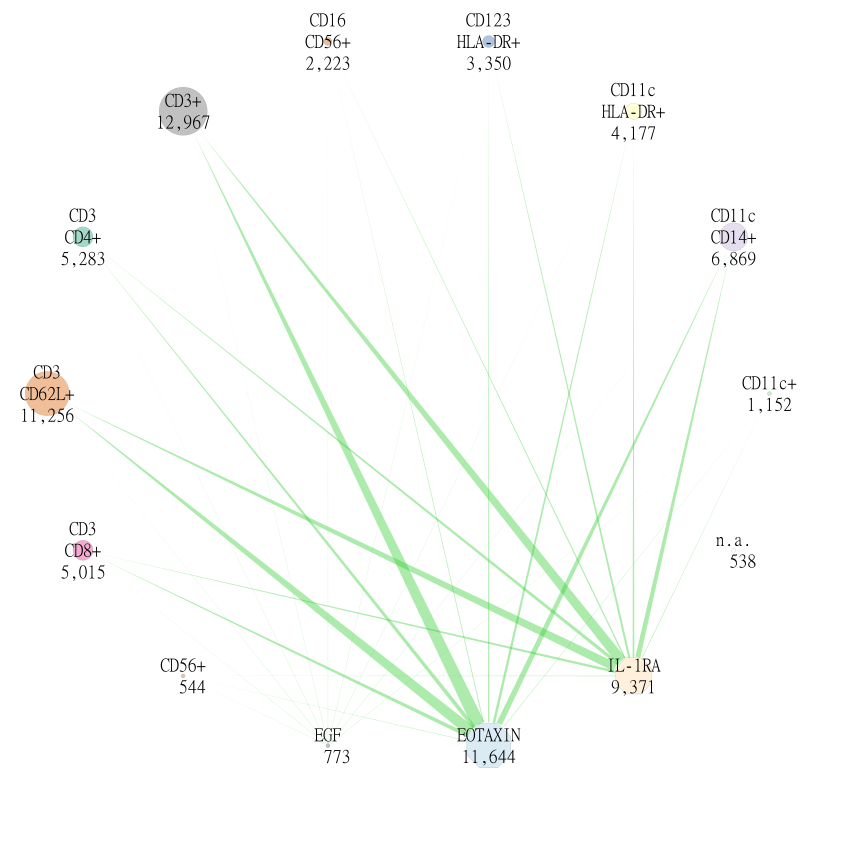

Healthy Patient 15: Female, age 29
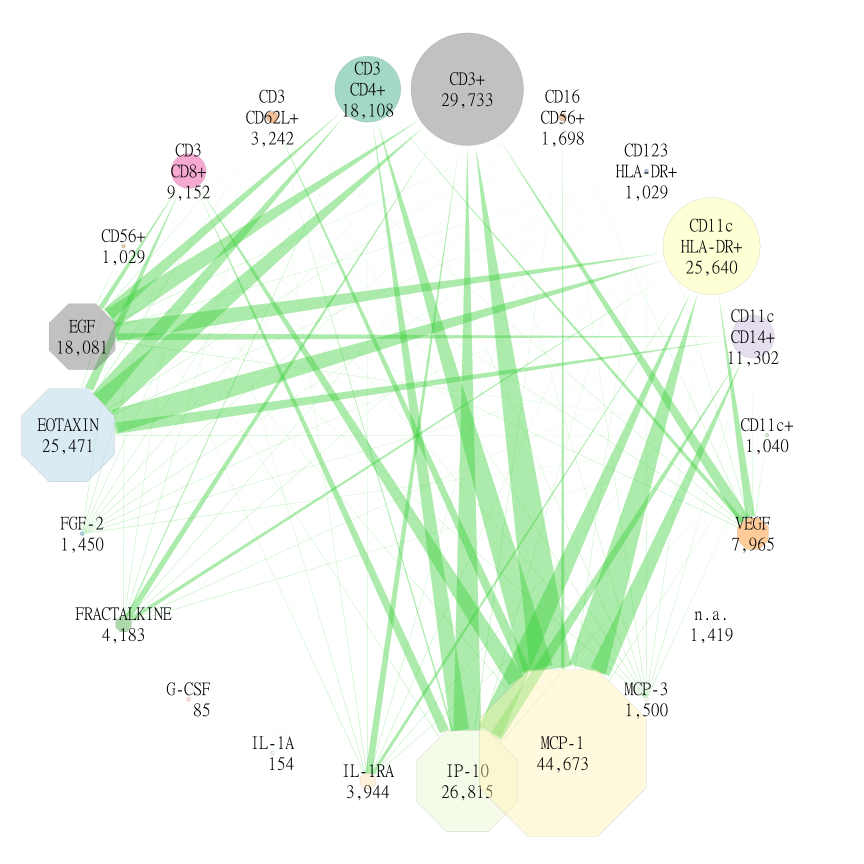

Healthy Patient 17: Male, age 26
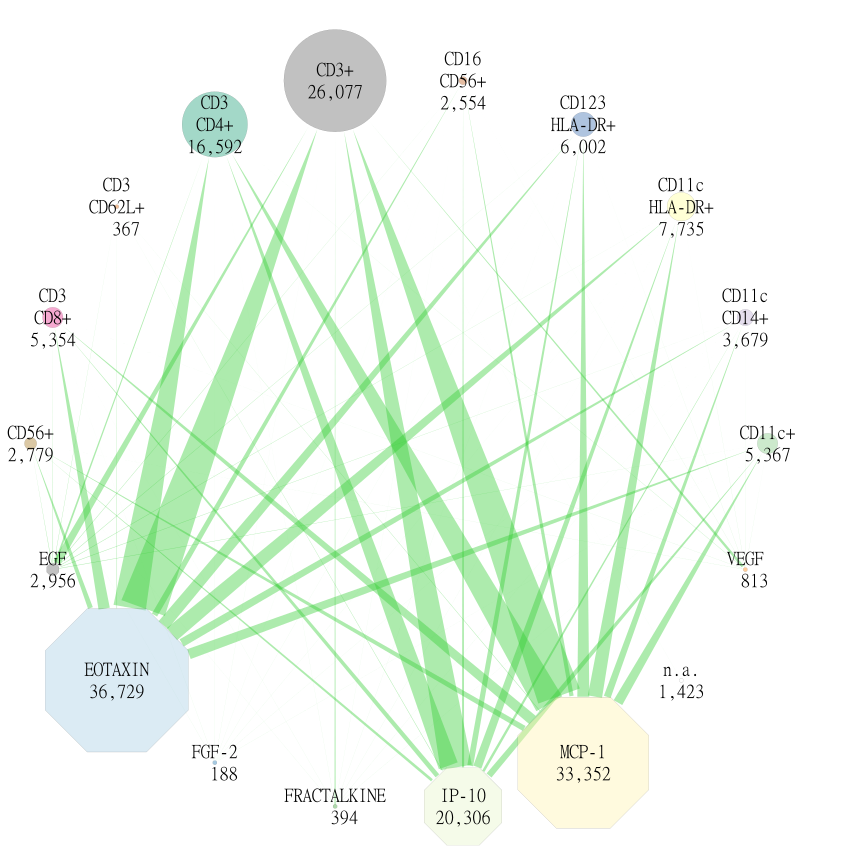

Healthy Patient 18: Female, age 24
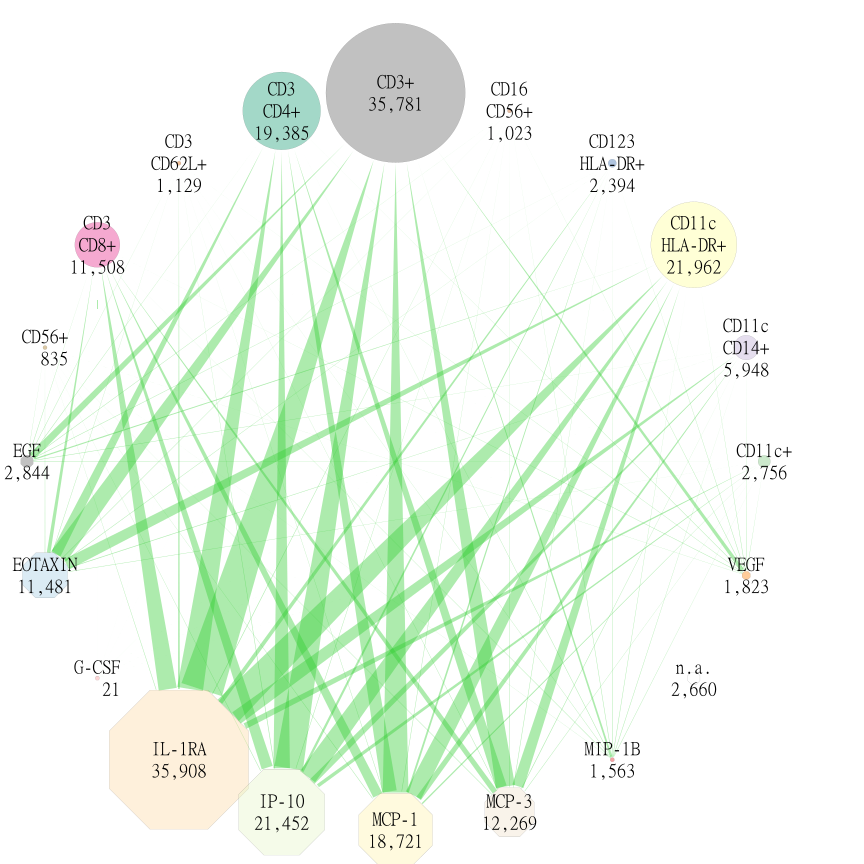

Healthy Patient 19: Female, age 23
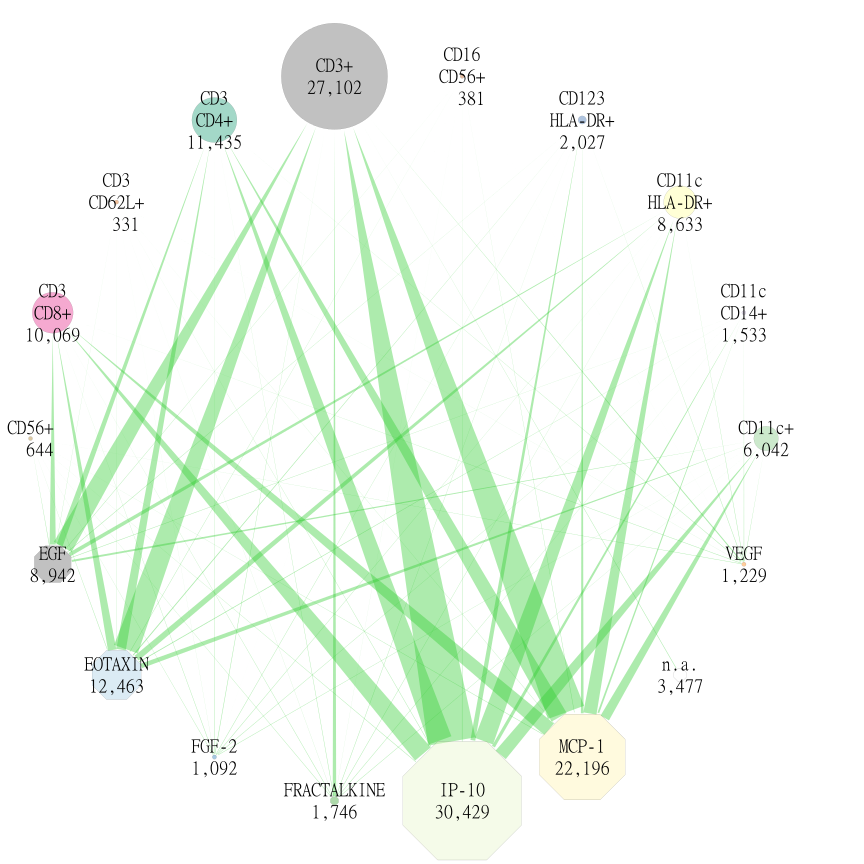

Healthy Patient 21: Female, age 29
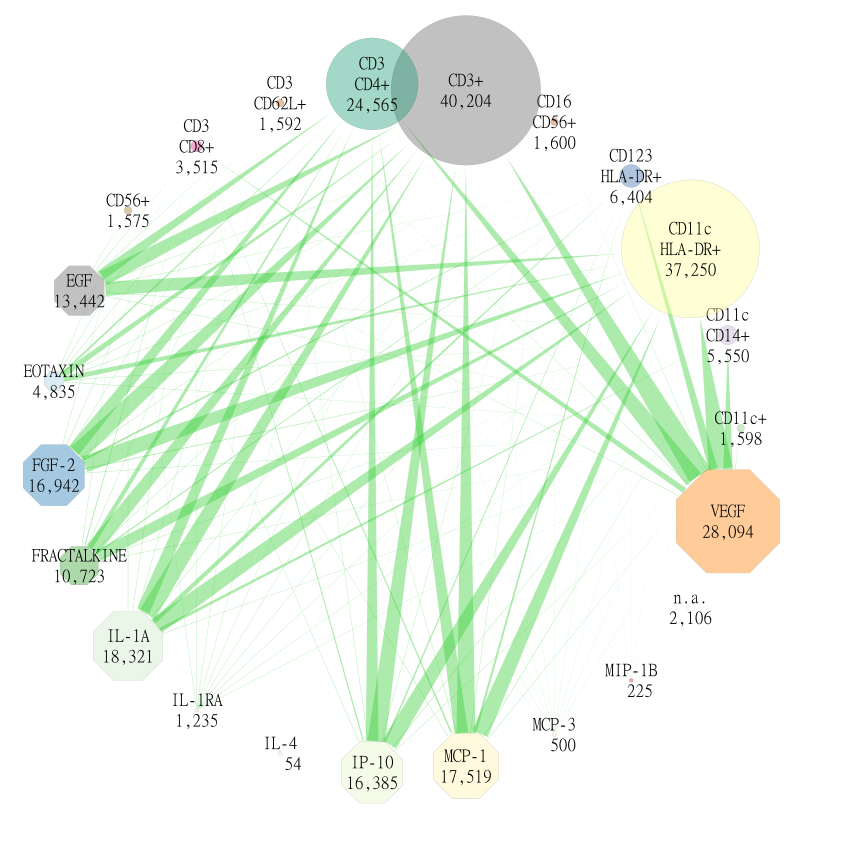

Healthy Patient 22: Male, age 33
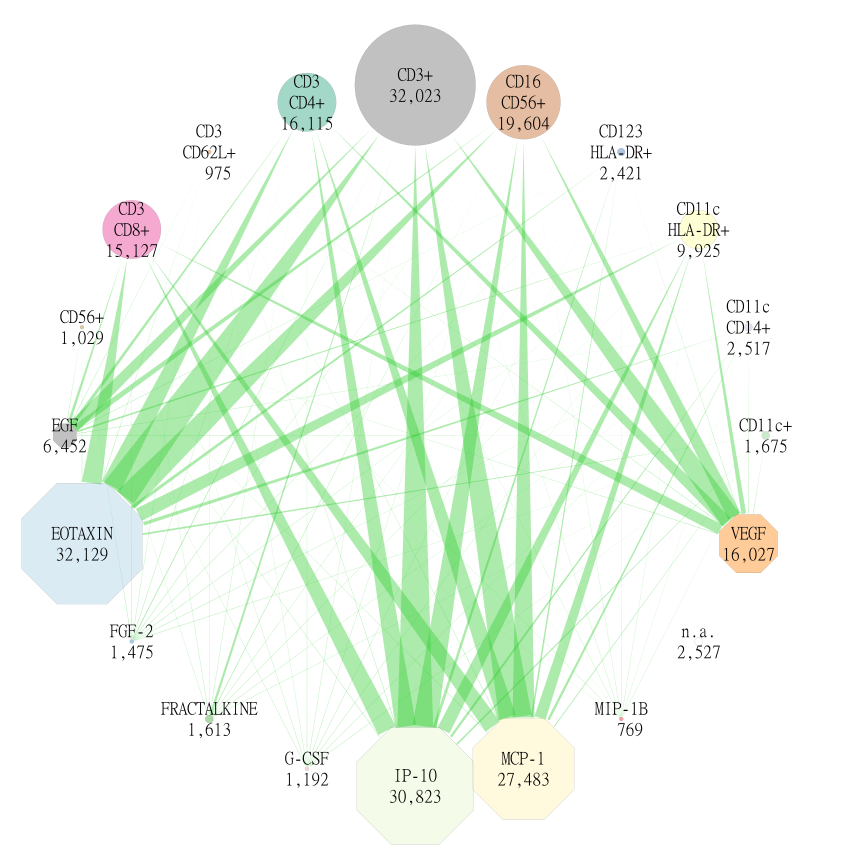

Healthy Patient 23: Male, age 30
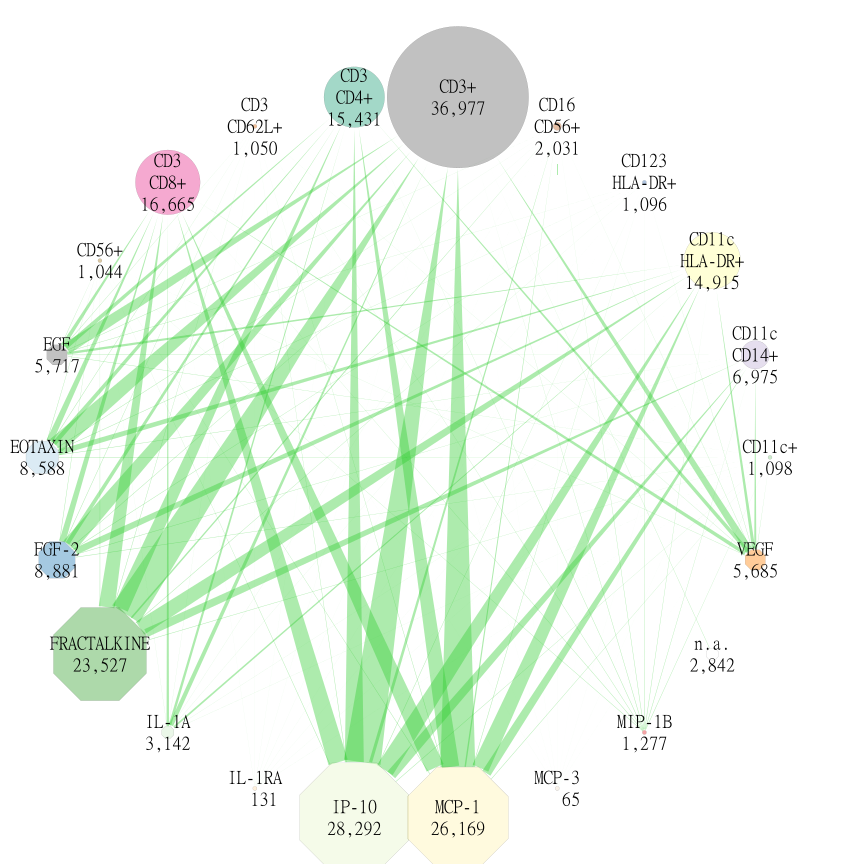

Healthy Patient 24: Male, age 34
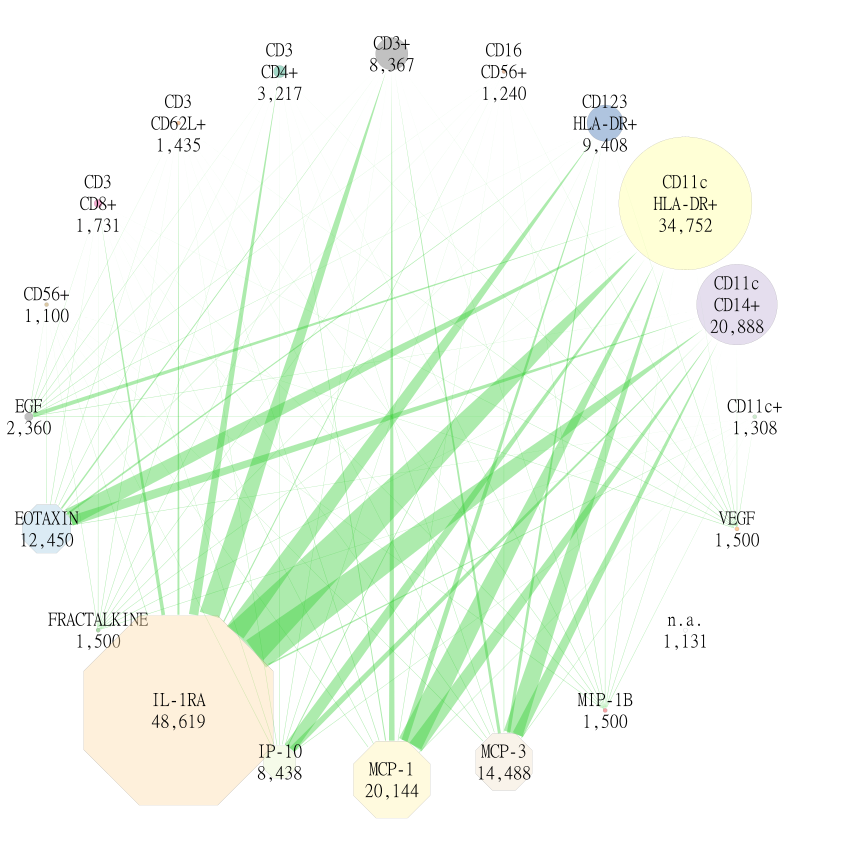

Healthy Patient 25: Female, age 22
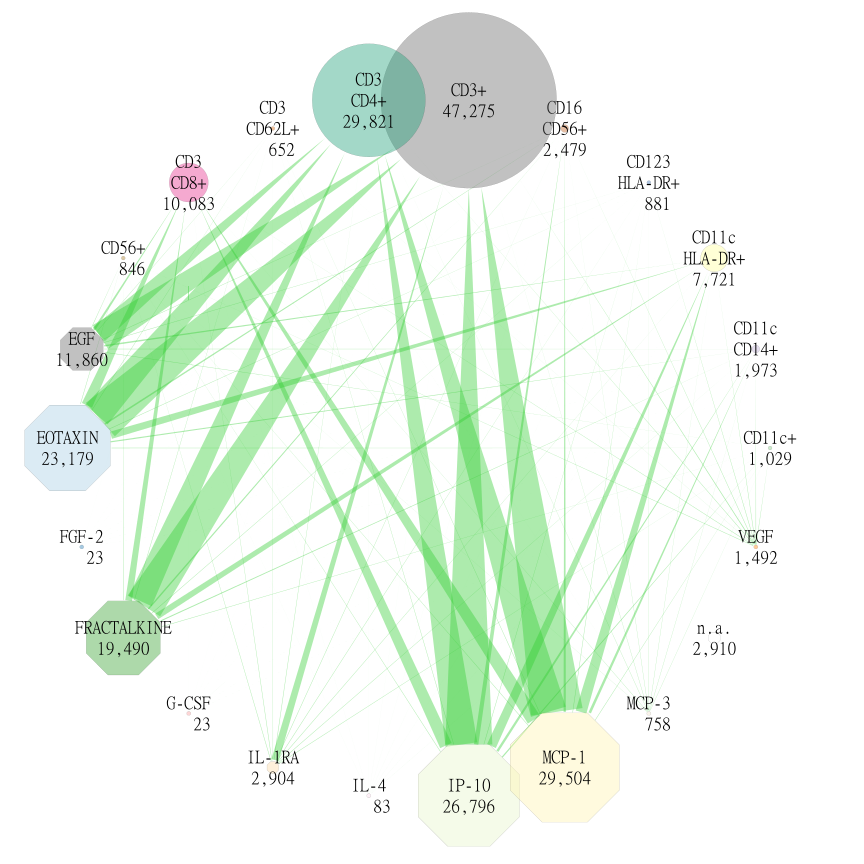

Healthy 26 Patient: Female, age 26
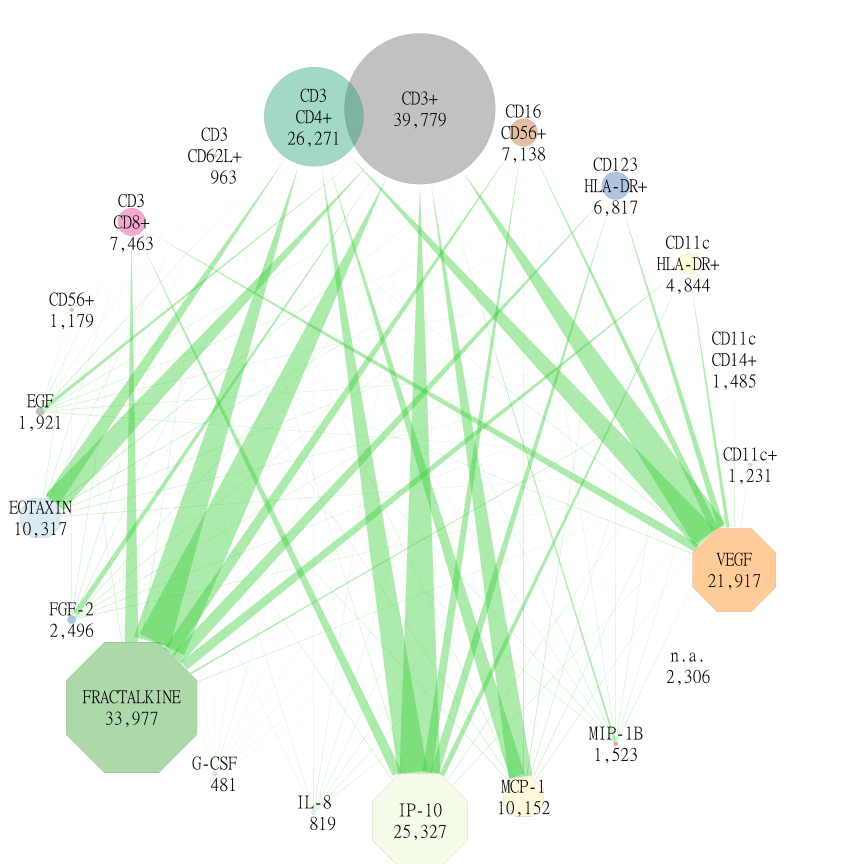

Healthy Patient 27: Male, age 19
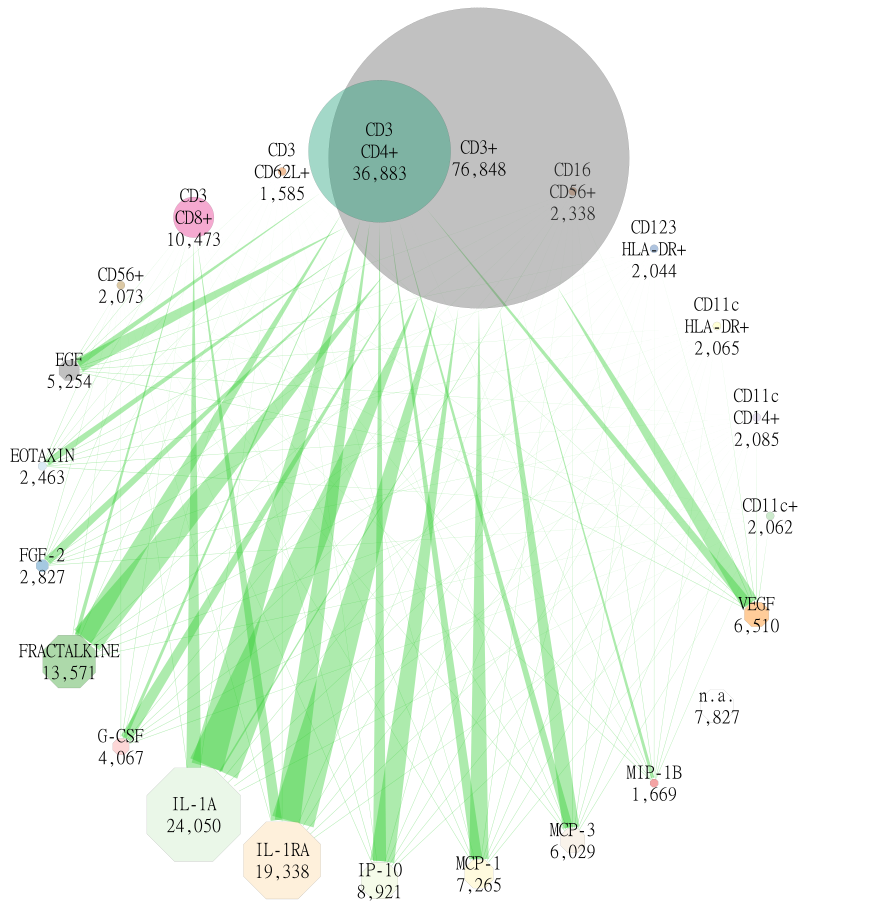

Healthy Patient 28: Female, age 32
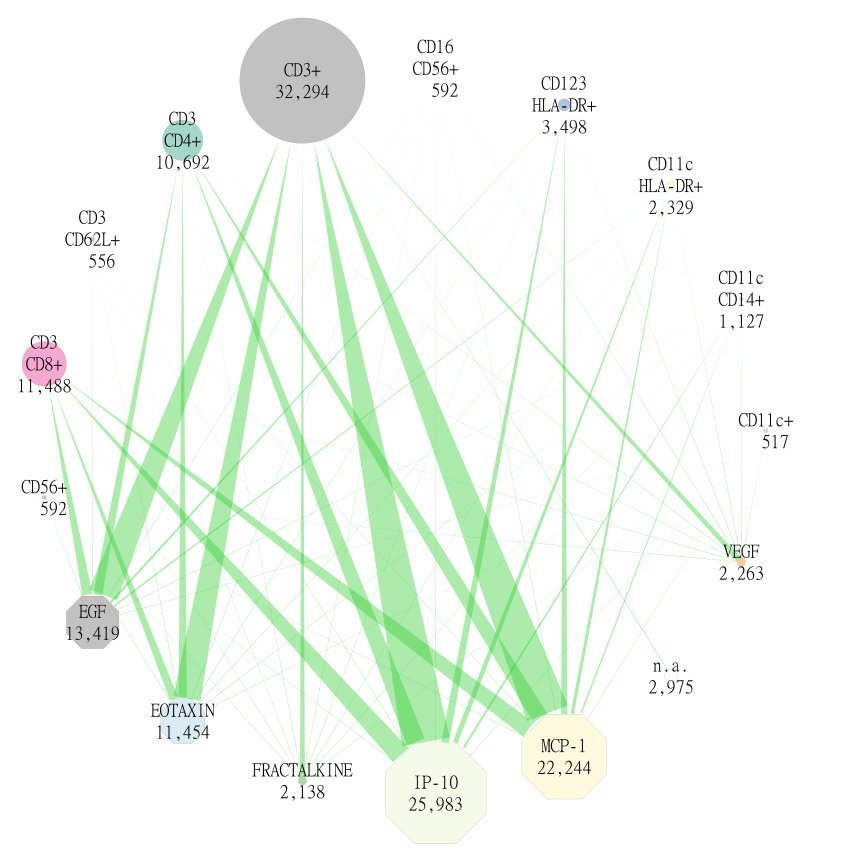

Healthy Patient 29: Male, age 22
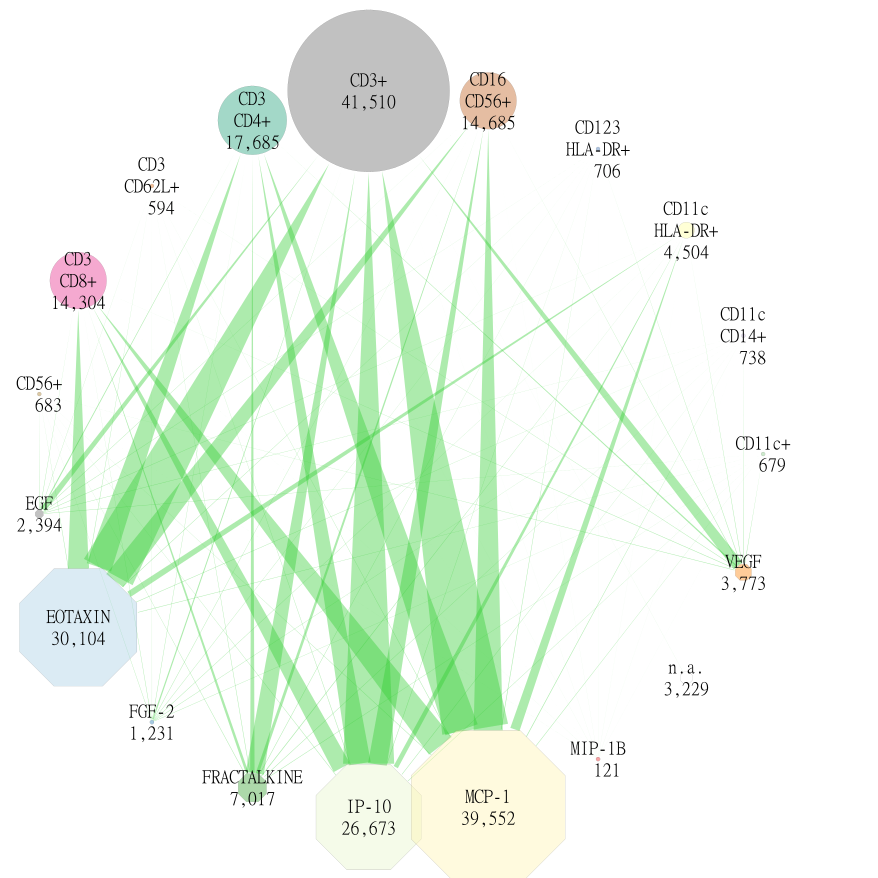

Healthy Patient 30: Male, age 30
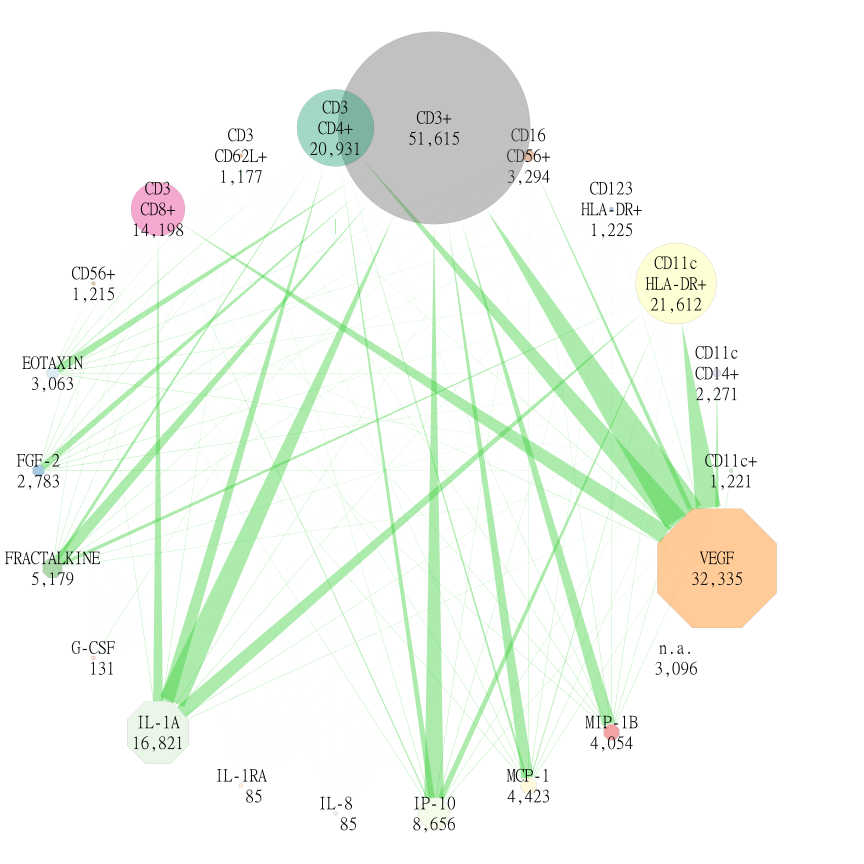

Healthy Patient 31: Female, age 33
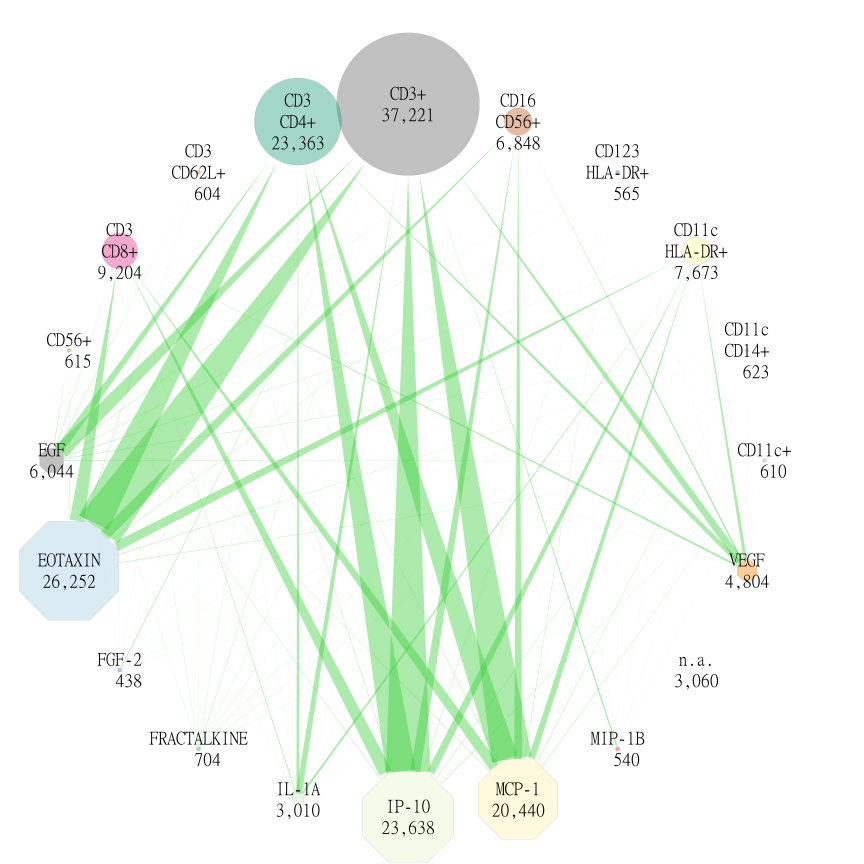

Healthy Patient 32: Female, age 25
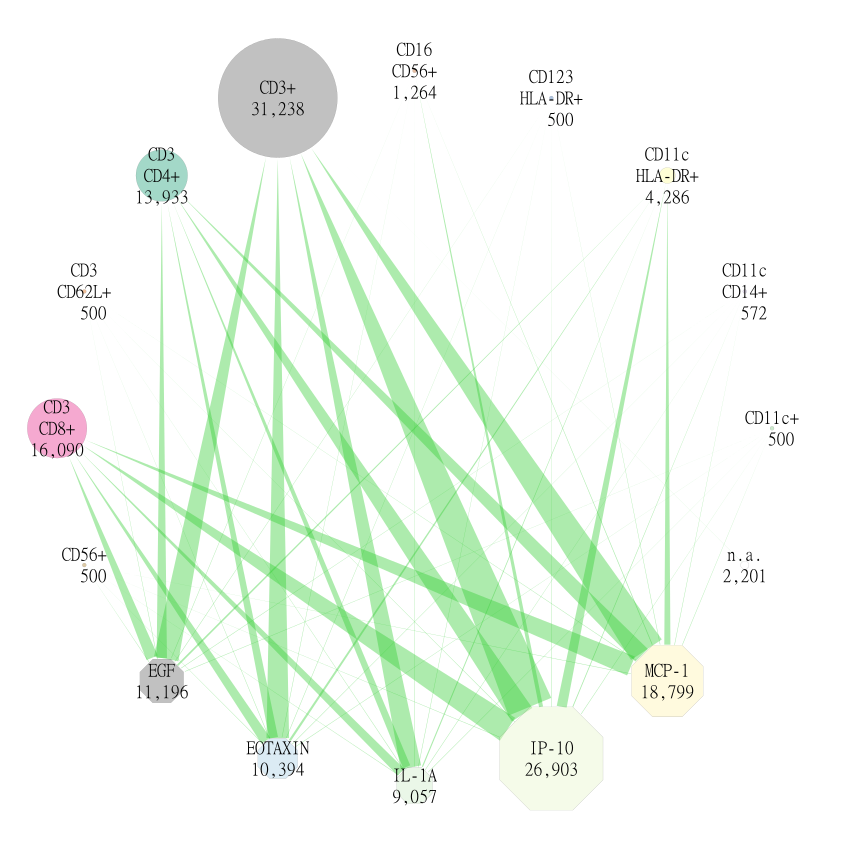

Supplement: Supplementary file 2 — Additional file 2. Individual Peripheral Blood Derived Serial Biomarker Data and Flow Diagrams: A graph of peripheral blood biomarker measurements and flow diagram is provided for each cancer patient and each healthy volunteer. All Quads for the individual are included in each diagram. Graphs are in the same format as Fig. 4. Flow diagrams are in the same format as Fig. 6. [file 12859_2021_4025_MOESM2_ESM.docx]
